# Stitching competition with digital threads: Unveiling the drivers of competitive success in the apparel sector

**DOI:** 10.1371/journal.pone.0325945

**Published:** 2025-06-10

**Authors:** Emmanuel Susitha, Amila Jayarathne, H. M. R. P. Herath

**Affiliations:** 1 Faculty of Graduate Studies, University of Kelaniya, Kelaniya, Sri Lanka; 2 Faculty of Management Studies and Commerce, University of Sri Jayawardanapura, Nugegoda, Sri Lanka; 3 Department of Information Management, SLIIT Business School, Malabe, Sri Lanka; University of Jaen: Universidad de Jaen, SPAIN

## Abstract

This study explores and validates the dimensions of digital capabilities and competitive performance within the apparel industry, aiming to develop a robust, multidimensional measurement instrument. Employing a sequential QUAL→QUAN exploratory design, the research began with in-depth interviews with key industry experts to identify critical constructs. These insights informed the subsequent quantitative phase, in which exploratory factor analysis and confirmatory factor analysis were applied to confirm the factor structure and validate the instrument. The study identifies four key dimensions of competitive performance in the apparel supply chain: customer satisfaction, better utilisation of resources, collaboration to compete, and strategic advantage. Process and technical digitalisation emerged as essential components of digital capabilities, reflecting the dual role of digital infrastructure and operational integration in enhancing performance. Theoretically, this research contributes by introducing validated instruments grounded in the Resource-based view, the Dynamic capabilities view, and the Extended resource-based view, offering an empirical framework that links digital capabilities with competitive performance. Practically, the instrument provides apparel manufacturers with a diagnostic tool to assess digital maturity and strategically align digital initiatives with performance goals. These findings are particularly relevant for firms navigating rapid technological change and seeking sustained global apparel supply chain competitiveness.

## Introduction

Globalisation and technological advancements have fundamentally transformed competition across industries. Organisations prioritise innovation, brand differentiation, and strategic resource management to remain competitive in market relevance and success [1]. In this evolving global market, competition has shifted from focusing on price and scale to a more complex dynamic where agility, speed to market, and innovation are critical determinants of competitive advantage [2]. Organisations must constantly innovate their product offerings and operational strategies to outperform competitors in fast-paced and ever-changing business environments.

Competitive performance (CP) has become a central measure of organisational success in this rapidly changing environment. CP reflects an organisation’s ability to outperform its competitors, ensuring long-term market relevance and profitability [3–6]. Research shows that companies with intense CP are better positioned to navigate market fluctuations, seize emerging opportunities, and effectively manage challenges [4,5]. This phenomenon is particularly relevant in industries with standard fast-paced change and high demand volatility. In such environments, businesses must constantly adapt, innovate, and make strategic decisions. These factors are essential in industries with short product lifecycles, rapid technological advancements, and intense competition [7–9]. Organisations in these environments face constant pressure to reduce time to market, innovate their product offerings, and manage costs while delivering high-quality service [10]. Balancing these demands and aligning them with long-term strategic goals is crucial for maintaining a competitive edge.

The apparel industry is one of the world’s most resilient sectors. Historically, it played a central role in industrialisation during the 1960s and 1970s, particularly in developed economies such as the United States, the United Kingdom, Japan, and various countries within the European Union, as well as in emerging markets [11,12]. However, starting in the 1980s, the global apparel industry experienced a significant transformation, shifting production to developing nations in Asia and Africa. Countries like China, Bangladesh, India, and Vietnam became major apparel exporters, leveraging lower labour costs and increasingly efficient supply chains to compete globally [13]. This shift has had profound socioeconomic impacts, creating millions of jobs, particularly for women, and helping many developing nations transition from agriculture-based economies to industrialisation. The apparel sector has played a vital role in reducing poverty and fostering socioeconomic growth in these countries by driving economic development [14]. As globalisation deepened, competition in the apparel industry intensified significantly. This heightened competition has forced leading companies to focus on critical areas such as innovative design, brand differentiation, and strategic marketing to maintain their competitive edge [1]. The fast fashion business model, characterised by rapid product turnover and constant consumer demand for new styles, has further accelerated competition, pressuring companies to continuously innovate in product development and supply chain efficiency [15].

The apparel industry is experiencing a significant transformation driven by the integration of digital technologies, which is crucial in enhancing competitive advantage and performance. Digital orientation and capabilities have positively impacted innovation and overall firm performance [16], with artificial intelligence becoming increasingly vital for integrating digital intelligence into core operations [17]. In fashion SMEs, digital capabilities (DCs) link strategic assets and competitive advantage, influencing firm performance [18], while IT assets are essential for improving organisational capabilities and operational efficiency [19]. Additionally, marketing capabilities, especially in image differentiation and promotions, are critical for maintaining competitiveness in fashion retailing [20]. Enterprise IT applications strengthen cross-channel capabilities and enhance managerial actions, improving operational efficiency and responsiveness to market demands [21].

Furthermore, supply chain capabilities, particularly those involving organisational culture and relational factors, contribute to operational performance and technology adoption. Sustainable technology adoption, driven by stakeholder pressures, enhances environmental and financial performance [22], while the digital revolution in enterprise architecture has improved operational efficiency and product innovation [23]. Despite the evidence supporting the role of digital technologies, there remains a gap in understanding which specific DCs most effectively contribute to CP in the apparel industry, particularly in developing economies [24].

At the same time, Digital technologies are crucial in enhancing business competitiveness and performance across various industries. Numerous studies have developed instruments to measure the impact of DCs, with a focus on areas such as digital supply chain activities [25], new product performance [26], overall business performance [27], sustainable competitive advantage [28], firm performance in international markets [29], and evaluating competitive capacity of business entities [30]. While these instruments provide valuable insights into specific aspects of digital transformation, there remains a significant gap in measuring the CP of apparel organisations, particularly concerning how DCs enhance their CP. Despite the apparel industry being a critical sector that has embraced digital transformation, instruments tailored to measure the unique competitive dynamics in this industry are lacking. Existing measurement tools are often generalised across industries, missing the apparel-specific factors like fast fashion dynamics, rapid product turnover, and sustainability pressures that uniquely shape competitiveness in this sector. Several scholars have pointed out that industry-specific instruments are necessary to accurately assess how digital technologies influence CP [22,23,31,32]. This lack of tailored instruments hinders the ability of apparel companies to systematically assess and leverage their DC for CP, underscoring the need for further research and development of specific tools. To address this empirical gap, the research objectives (ROs) were established:

RO1: To explore the DC dimensions and indicators that impact the CP of the apparel industry.
RO2: To explore the CP dimensions and indicators of the apparel industry.
RO3: To develop the measurement scales for CP and DC of the apparel industry.

The remainder of this paper includes a comprehensive literature review, followed by detailed data analysis, discussion of the findings, and concluding remarks.

## Literature review

This literature review provides a concise overview of DCs and CP, laying the foundation for the research and contextualising its key themes.

### Theoretical influence

To understand how digital transformation drives competitive performance in the apparel supply chain, it is insufficient to rely on a single theoretical lens. Traditional theories often fail to explain the complex, evolving role in volatile and highly competitive environments. This study argues for an integrated theoretical foundation drawing on the Resource-Based View (RBV), the Dynamic Capabilities View (DCV), and the Extended Resource-Based View (ERBV) to more comprehensively explain how digital transformation contributes to sustainable competitive advantage in the apparel industry.

The RBV has long been a dominant framework in strategic management, asserting that firms gain sustainable competitive advantage by leveraging resources that are valuable, rare, inimitable, and non-substitutable [33]. While this view offers a useful starting point, it is limited in its ability to account for rapid technological change and digital disruption. Nonetheless, when embedded in firm-specific routines and integrated across processes, digital capabilities meet the VRIN criteria [34]. In the apparel supply chain, digital planning systems, real-time data analytics, and supply chain automation can no longer be considered generic IT tools; they are now strategic resources that shape cost structures, customer responsiveness, and production flexibility [35]. Therefore, this study contends that RBV remains relevant, but must be adapted to recognise the strategic heterogeneity and intangible nature of digital capabilities in contemporary supply chains. While RBV focuses on resource possession, it fails to address how firms respond to rapid environmental change. In highly dynamic markets such as apparel, where demand volatility, fashion cycles, and customer preferences shift rapidly, the ability to reconfigure and adapt resources becomes paramount. Here, the DCV offers a more appropriate lens. Competitive advantage lies not only in what a firm owns but in what it can do with those assets in turbulent environments [36–39]. This study posits that digital capabilities are dynamic mechanisms that allow firms to sense market changes, seize new opportunities, and transform operational processes. Technologies such as AI-based forecasting tools, digital sampling platforms, and collaborative supply chain portals exemplify how firms adapt proactively to shifting demands. In line with Eisenhardt and Martin [40], this view is especially relevant for firms in fast fashion, where speed and flexibility are not just operational advantages but survival imperatives.

The RBV and DCV, however, remain largely inward-looking. Such internal focus is inadequate in today’s digitally interconnected and sustainability-conscious global supply chains. The ERBV offers a necessary extension by recognising the role of externally embedded resources, particularly those related to environmental sustainability, inter-firm collaboration, and technological ecosystems [41,42]. This study argues that the value of digital capabilities cannot be fully understood without considering their role in shaping external relationships and stakeholder alignment. Tools such as blockchain for supply chain traceability, IoT-enabled monitoring for compliance, and cloud-based platforms for supplier integration illustrate how digital transformation extends beyond firm boundaries to influence ecosystem-wide competitiveness [43,44]. ERBV thus enables a broader understanding of how digital strategies contribute to firm-level performance, long-term resilience and value co-creation across the supply chain. RBV, DCV, and ERBV offer complementary yet distinct insights into how digital capabilities influence competitive performance. This study argues that relying on any single framework would provide an incomplete picture. The RBV explains the foundational value of digital capabilities, DCV captures their dynamic and adaptive nature, and ERBV situates them within broader network and sustainability contexts. By integrating these perspectives, this study develops a theoretically rigorous foundation to assess how digital transformation can enable apparel firms to achieve sustained competitiveness in a digitally evolving global marketplace when measured and aligned strategically.

### Competitive performance in the apparel industry

CP in the apparel industry is vital for sustaining a competitive edge in today’s highly dynamic and fast-paced global market. Unlike other industries where internal operational efficiency might suffice, in the apparel sector, the performance of the entire supply chain (SC) from delivering products on time to maintaining availability and demonstrating agility directly influences a company’s competitiveness [45]. CP in the apparel industry is not just about individual firms excelling but about how effectively the extended SC, including suppliers, manufacturers, logistics providers, and retailers, works together to meet end customers’ expectations. Achieving this comprehensive performance is crucial for companies to succeed in an industry marked by short product life cycles, fluctuating demand, and globalised production networks.

A central aspect of CP in the apparel industry is operational performance, which encompasses critical metrics such as cost control, adaptability, efficiency, and timely delivery [46]. Unlike more generic supply chains, apparel companies must be highly flexible to adapt to seasonal shifts, changing fashion trends, and evolving consumer preferences. In this context, agility becomes a critical determinant of CP, allowing the SC to quickly respond to market disruptions such as sudden changes in demand or supply shortages [47]. Apparel companies that fail to embed agility in their supply chains risk losing market share to competitors who can respond more quickly and efficiently. Therefore, CP in this sector is closely linked to the SC’s ability to adjust production and distribution in real-time to align with market needs, making agility a key factor for gaining a competitive advantage. Integrating operational and financial metrics in evaluating CP is particularly important in the apparel supply chain. While economic performance indicators such as profitability and return on investment remain essential, an accurate measure of CP goes beyond these financial outcomes. Operational excellence, characterised by reduced lead times, optimised production schedules, and efficient resource utilisation, directly impacts a supply chain’s ability to drive profitability [48]. For apparel supply chains, this requires balancing cost-efficiency with meeting tight deadlines and maintaining product quality. The unique challenges of the apparel industry, such as rapid changes in fashion and the complexity of global distribution, demand that supply chains focus on both financial results and operational flexibility, making CP a multidimensional concept that requires attention to both areas [49].

Additionally, relational capabilities are critical in enhancing CP within the apparel industry. Strong partnerships between suppliers, manufacturers, and retailers ensure smooth coordination across the entire SC. Khai, Mohamed, and Hassan [50] emphasise that building supplier partnerships, maintaining strong customer relationships, and facilitating efficient information sharing are essential for improving CP. These relationships are critical in the apparel industry, where production frequently spans multiple countries and requires tight synchronisation among various stakeholders. For instance, aligning production schedules between suppliers and manufacturers can significantly reduce lead times, enhance responsiveness, and lower costs, which are crucial factors for maintaining CP. Sharing real-time information across the SC allows companies to mitigate risks and make agile decisions, which is especially important in a volatile market environment. Efficiency within the apparel industry’s SC is another major factor influencing competitive performance. Ahmad, Khalil, and Rashed [51] suggest an integrated SC model that optimises resources and minimises waste. This approach is crucial in an industry with slim profit margins, and speed is a critical differentiator. Integrating various stages of the SC, from raw material sourcing to final product delivery, ensures resources are utilised efficiently and that the SC operates without unnecessary delays or costs. This integration is critical to maintaining CP, as it enables companies to reduce operational costs while enhancing service delivery, ultimately leading to greater profitability and competitiveness in the market.

### Competitive performance indicators of the apparel industry

One of the essential indicators of competitive performance is maintaining a competitive unit price, which plays a pivotal role in attracting and retaining customers [52,53]. Closely linked is the continual optimisation of pricing structures to better serve customers, allowing companies to remain competitive in a fluctuating market [52,53]. Responsiveness to market dynamics and emerging opportunities is another critical factor, as firms must adapt quickly to changes in demand and external conditions. This responsiveness is further enhanced by the agility to swiftly adjust to evolving market conditions, enabling companies to stay ahead of competitors [54,55]. Improving production lead times is another essential aspect, as reducing lead times allows firms to meet customer demand faster and more efficiently [54]. Timely product delivery that outpaces competitors is a crucial indicator, ensuring that customers receive products faster, which enhances customer loyalty and market positioning [55–58]. Furthermore, leveraging information systems to monitor all operational aspects meticulously ensures better control and transparency across the supply chain. Real-time data tracking across supplies, finished goods, equipment, and personnel provides businesses with critical insights that allow for more agile decision-making and operational efficiency [54].

Flexibility in adjusting the product mix according to market demands is another vital indicator, allowing firms to adapt their offerings to meet the shifting preferences of consumers [55,59]. A diverse range of products tailored to varied customer preferences further strengthens competitive performance by broadening market reach [53,60,61]. Additionally, meeting customer specifications for product features and technical requirements is essential for maintaining satisfaction and fostering repeat business. To ensure the highest standards, rigorous quality processes are necessary to guarantee product excellence, a key driver of long-term competitiveness [62]. Better utilisation of resources is also central to competitive performance. Maintaining minimal inventory levels of raw materials and finished goods is vital for streamlining operations, reducing waste, and improving efficiency. Equally important is optimising production lines to ensure a balance between efficiency and flexibility, which contributes to operational excellence [63,64]. Compared to industry peers, companies that maintain low unit manufacturing costs are better positioned to compete on price while maintaining profitability. Additionally, achieving high on-time delivery performance is critical to meeting customer expectations and maintaining trust [55,57,59].

Collaboration within the supply chain is another key driver of competitive performance. Sharing proprietary information and communicating any changes that may impact supply chain partners enhances transparency and trust, which are critical for effective collaboration. Supplier participation in new product development and design processes fosters innovation and strengthens relationships, leading to more responsive and adaptive supply chains [54,55,59]. Information exchange among suppliers, customers, and other external stakeholders is essential for fostering collaboration and ensuring all parties are aligned in their goals [54]. Information sharing facilitates collaboration and improves overall supply chain efficiency [55,65,66]. Transparent data flows between supply chain members enable collaborative decision-making, which is crucial for responding to market changes and ensuring operational agility. Collaborative demand forecasting techniques further enhance coordination, allowing supply chain partners to optimise production and inventory planning, which leads to better outcomes across the supply chain [54]. Strategic advantage is a final critical area of competitive performance. High flexibility in adjusting manufacturing volume to meet market demands allows companies to respond quickly to shifts in demand, ensuring that they can capitalise on market opportunities without incurring unnecessary costs [55,59]. This flexibility is a significant competitive advantage, especially in industries like apparel, where consumer preferences can change rapidly.

In the context of competitiveness in the apparel industry, customer satisfaction remains the cornerstone for maintaining a market presence and driving business growth. The literature consistently highlights the importance of understanding and responding to customer needs, emphasising that it is advantageous and essential for survival in this highly competitive sector [67–71]. Better resource utilisation is another critical factor in achieving sustainable competitive advantage, outlined by Kleindorfer, Singhal, and Van Wassenhove Kleindorfer, Singhal [72], who advocate for optimised resource management to gain a competitive edge. The variation observed across interviews suggests strategic differences in resource management approaches, which align with the firm’s resource-based view. This perspective posits that companies can leverage unique configurations of resources to achieve and maintain competitive positioning [33]. While collaboration is recognised as a strategy to enhance competitiveness, as noted by Simatupang, and Sridharan Simatupang and Sridharan [73], its limited implementation may reflect the complexities inherent in fostering collaborative efforts within diverse supply chains. The literature underscores the necessity of integrating information and collaboration to improve product quality and reduce costs. However, the challenges of coordinating across different cultural and business environments persist [74–76]. Strategic approaches and innovation highlight the need for clearly defined strategies and ongoing innovation as critical components of competitive strategy, as Porter [77] emphasises. The relatively low emphasis on these themes suggests a gap between recognising their importance and actual implementation, a challenge frequently discussed in the literature on SC management [78]. The underemphasis on adaptability and vertical integration also indicates potential vulnerabilities. As Shukla, and Sushil Shukla and Sushil [79] argue, adaptability is vital for sustaining competitiveness in volatile markets, while vertical integration can enhance efficiency and performance in apparel SCs [80].

### Digital capabilities of the apparel industry

The apparel industry is undergoing a profound digital transformation, with information technology playing a critical role in shaping organisational capabilities and enabling cross-channel strategies that enhance competitiveness and operational efficiency [19,81]. Integrating digital technologies such as artificial intelligence, big data analytics, and the Internet of Things is revolutionising business practices across the supply chain, from product design to distribution. AI, for instance, is being leveraged to analyse consumer data, predict fashion trends, and optimise inventory management, reducing costs and improving responsiveness to market demands [23]. Big data analytics is helping companies gain deeper insights into consumer behaviour, enabling more personalised marketing strategies and product offerings. One of the most significant developments in the apparel industry is the shift towards mass personalisation. Utilising technologies like 3D avatars and virtual prototypes, companies can offer customised products that cater to individual consumer preferences, creating a more engaging and tailored shopping experience [82]. This approach enhances customer satisfaction and reduces waste and production inefficiencies, aligning with sustainability goals. Moreover, digital printing technologies are expanding creative possibilities in apparel design, allowing for greater flexibility in production and faster response times to fashion trends [83]. Adopting these technologies has facilitated the rise of on-demand production models, which are more agile and responsive to fluctuations in consumer demand.

Retailers are also increasingly adopting various digital platforms to optimise operations and improve the customer experience. For example, lifestyle apparel retailers integrate e-commerce solutions with brick-and-mortar operations, utilising omnichannel strategies to provide seamless shopping experiences across multiple touchpoints [84]. Technologies like augmented reality and virtual reality enhance the online shopping experience, allowing customers to try on clothes before making a purchase virtually, thereby reducing return rates and improving customer satisfaction [85]. However, the pace of digitalisation varies significantly across regions, with some countries still transitioning from Industry 3.0 to Industry 4.0 [86]. Developed economies are often at the forefront of adopting advanced digital technologies, while emerging markets face challenges such as limited infrastructure, skills gaps, and resistance to change [87]. Despite these challenges, digital transformation is essential for companies to remain competitive in the rapidly evolving apparel industry. Companies that fail to embrace these technologies risk falling behind as the industry continues to prioritise speed, efficiency, and customisation.

Digitisation has transformed the apparel industry by streamlining operations, enhancing supply chain visibility, and enabling data-driven decision-making. Technologies such as product lifecycle management, digital colour management, and 3D virtual prototyping have significantly reduced time-to-market and minimised resource wastage in product development processes [88]. Moreover, digital supply chain platforms and AI-driven demand forecasting tools have enhanced agility, allowing manufacturers to respond more swiftly to market fluctuations and consumer preferences [89]. This digital shift improves operational efficiency and supports sustainability goals by enabling better planning and reducing overproduction. The emergence of the metaverse further extends the frontiers of digitisation by introducing immersive consumer experiences and new business models. In virtual environments, fashion brands can create interactive showrooms, conduct digital fashion shows, and sell virtual garments through NFTs (Non-Fungible Tokens), expanding their presence beyond physical constraints [90]. This enables brand differentiation and engages digitally native consumers who value personalised, interactive experiences. For example, platforms like Decentral and Roblox have become spaces where fashion brands such as Gucci and Nike experiment with digital-first product launches and collaborations [91]. From a manufacturing standpoint, the metaverse and extended digital ecosystems facilitate virtual sampling, remote collaboration, and 3D design integration, reducing the need for physical samples and international travel. These advancements enhance speed, reduce cost, and contribute to environmental sustainability. As the apparel industry becomes increasingly digital, the convergence of AI, virtual reality, and blockchain is expected to redefine value creation across the fashion supply chain, from product ideation to post-purchase engagement. Embracing these innovations enhances competitiveness and positions firms to thrive in a rapidly evolving digital economy.

### Digital capabilities vs competitive performance

The importance of DCs in today’s trade and economic environment cannot be overstated. DC enables businesses to efficiently disseminate information, innovate in product and service design, identify new commercial opportunities, and develop adaptable and innovative corporate strategies [92]. Moreover, DC is a critical tool for enhancing CP in an ever-changing landscape [93,94]. As noted by Bhatt and Grover [93], modern IT technologies empower businesses to respond to threats and capitalise on opportunities quickly. This organisational capability encompasses various activities, including searching, acquiring, assimilating, and applying resource knowledge and identifying and orchestrating opportunities that lead to a competitive advantage [93,95].

In another study, Dehning and Stratopoulos Dehning and Stratopoulos [96] empirically investigated the factors contributing to sustained CP through implementing DC strategies. Their research highlighted a strong correlation between managerial DC skills and long-term sustainability. However, the study found that technical DC expertise or infrastructure alone was not a source of enduring competitive advantage. Furthermore, Zhang, Lee and Zhao Lai, Zhang [97] examined the impact of DC on the CP of third-party logistics (3PL) companies in China, discovering that the nonlinear effects of digital technologies significantly influence a firm’s competitive edge. Okumus Okumus [98] also emphasises that businesses can sustain CP by leveraging DC to effectively create, store, transfer, and utilise implicit and explicit knowledge. Examining the use of DC in Turkish contractor organisations, Cakmak and Tas Cakmak and Tas [99] found that while most operational-level tasks using DT primarily have technical and economic implications, strategic applications of DC were limited. Although strategically employing DCs is essential for gaining a competitive edge, the evidence suggesting a significant enhancement in competitiveness remains scarce. However, the accompanying DC resource system undeniably affects a company’s competitive position in the market [100].

Effectively using DC within the SC reduces lead times, optimises capacity utilisation, and enables adaptability to new market dynamics and customer expectations. These dynamics and expectations enhance the SC’s ability to quickly respond to market threats and reconfigure resources as needed [101]. Additionally, trust among SC members amplifies the impact of DC-based SCA on firm performance, fostering collaborative efforts that reduce costs, improve product innovation processes, and ultimately deliver more excellent value to customers [102]. The broad adoption of DC enables SC actors to access and share vital information, which is essential for ensuring timely deliveries, increasing product customisation, enhancing customer satisfaction, and shortening product lead times. To maximise the benefits of DC deployment, management must support these efforts by providing employees with the necessary DT training. This will enhance the SC’s overall value generation potential and bolster their firms’ long-term competitiveness [103].

A lack of adequate measurement instruments hinders the CP of the apparel industry. Dinoka Nimali, Khatibi [104] identified significant gaps in employee-organisation congruence factors, indicating a need for better alignment in performance metrics. Narawita and Layangani [105] emphasised the importance of performance measurement systems in achieving organisational objectives. Many developing countries, including Sri Lanka, lack such systems. Perera and Perera [106] proposed a performance measurement system tailored for lean manufacturing environments, addressing the inadequacies of traditional metrics. Abeykoon and De Alwis [107] highlighted the impact of Total Quality Management practices on export performance, suggesting that quality measurement is crucial. Sivalogathasan [108] linked intellectual capital to innovation capability and organisational performance. Abeysekara, Wang [54] explored supply-chain resilience’s effect on performance, and Durairatnam, Chong [109] focused on quality performance determinants, underscoring the need for comprehensive measurement frameworks in the industry.

## Methodology

Pragmatism offers a solid philosophical foundation for a mixed-methods research strategy, combining qualitative and quantitative approaches. Scholars like Denscombe [110] and Mitchell and Education [111] describe pragmatism as the “philosophical spouse” of mixed research because its core principles allow for the integration of different methodologies. Pragmatism focuses on “what works,” emphasising addressing real-world problems through action-oriented research [112,113]. This flexible approach allows researchers to select the best qualitative or quantitative methods to answer research questions effectively. Mixed-methods research designs, such as exploratory sequential, involve qualitative research followed by quantitative validation. These designs are well-suited for investigating complex issues like DCs and CP in industries [114,115].

This study uses a mixed-methods approach to address the apparel industry’s ROs related to DCs and CP. The pragmatist paradigm underpins the methodology, allowing for the integrating of qualitative and quantitative methods. This combination capitalises on the strengths of both approaches while minimising their limitations. The exploratory sequential design (QUAL→QUAN) is employed, where qualitative methods are used to explore and identify critical dimensions, followed by quantitative methods to confirm and validate findings. This method aligns with the research goals of investigating how DCs impact CP [115]. Through this design, the study aims to explore, develop, and empirically test instruments to measure the DCs that drive competitiveness in the apparel industry.

### Research design

Semi-structured interviews were conducted as the primary data collection method, chosen for their flexibility in exploring participants’ insights while allowing follow-up questions to delve deeper into specific areas of interest [116]. A purposive sampling strategy was employed to ensure that participants had significant experience and knowledge in the apparel industry, making the data collected relevant and informative [117]. Participants included high-level professionals from various roles in the apparel industry, such as Chief Information Officers (CIOs), Chief Executive Officers (CEOs), and Vice Presidents (VPs). Interviews were audio-recorded with consent and transcribed verbatim to facilitate accurate data analysis.

Eight in-depth interviews were conducted using a semi-structured, open-ended interview guide, which was refined based on feedback from academic and industry experts. Please see Table 1 for interview details.

**Table 1:** Interview details.

Interview	Date	Designation	Industry Experience	Mode	Size (Employees)
INT - 1	07/08/2023	CIO	20 + years	Online – Zoom	60,000+
INT - 2	17/08/2023	VP – Sales	32 years	Online – Zoom	8500+
INT - 3	26/08/2023	CTO	30 + years	Online – Zoom	110,000+
INT - 4	18/09/2023	Director – Transformation	12 years	Online – Zoom	60,000+
INT - 5	18/09/2023	Director – Supply Chain	35 + years	Online – Zoom	60,000+
INT - 6	03/10/2023	CEO	40 + years	Online – Zoom	12,000+
INT - 7	27/10/2023	VP – Operations	27 years	Physical	8500+
INT - 8	12/11/2023	CEO	25 + years	Online – Zoom	20,000+

The interviews were conducted until data saturation was reached, ensuring no new information emerged from additional interviews. This study adopted inductive reasoning to develop context-specific conceptual frameworks that enrich understanding of business phenomena [118,119]. However, qualitative research faces challenges, such as ensuring sample representativeness and mitigating researcher bias [120]. To address these issues, we applied the trustworthiness framework proposed by Singh, Benmamoun, Meyr, and Arikan Singh, Benmamoun [121], focusing on four critical criteria: credibility, transferability, dependability, and confirmability. Two trained researchers conducted the coding process, achieving Krippendorff’s alpha of 0.88, indicating a high level of intercoder reliability and reinforcing the robustness of the data analysis process [122]. NVivo software was used to analyse the interview transcripts, facilitating the identification of recurring themes and patterns [123]. The initial coding process involved familiarising the data, generating codes and grouping these codes into broader themes. The iterative process of reviewing and refining themes ensured that they accurately represented the underlying data. This rigorous approach enabled the researchers to answer RO1 and RO2 to identify and define critical dimensions of CP and DCs in the apparel industry, providing a preliminary measurement instrument to analyse further [124].

**Fig 1:**
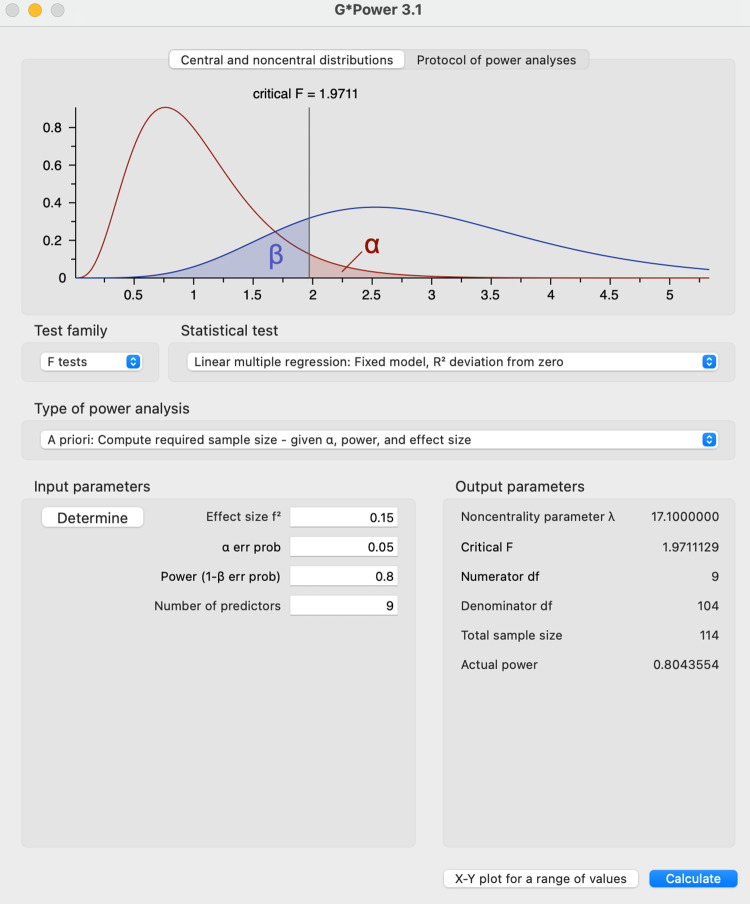
Power analysis of the sample model.

The quantitative phase was designed to address RO3 by developing a measurement tool. A sampling strategy is essential because collecting data from every unit within a population is impractical, and determining an appropriate sample size is crucial for drawing valid conclusions from research findings, despite being one of the most challenging aspects of designing empirical research [125–127]. Recent research studies and developments suggest that researchers should use power analysis [128–132] to determine the sample size, taking into account the section of the model with the most significant number of predictors [133,134]. This study analysed power using G*Power 3.1.9.7, a free and widely accessible software tool. With an effect size of 0.15 (medium effect), an alpha level of 0.05, and a power of 0.80, the required sample size for this study is *n = 114*. Fig 1 below shows the power analysis of the sample model.

Participants were recruited randomly through existing contacts to ensure a diverse and representative sample [135]. The inclusion criteria included executives, junior managers, senior managers, and directors/CEOs with experience in the apparel industry and supply chain management. Participation was entirely voluntary, with no incentives provided to ensure the integrity and authenticity of responses [136]. This approach aligns with ethical research practices that emphasise voluntary participation and the absence of coercion [137].

A cross-sectional study was conducted using a self-administered online survey developed through Google Forms. The survey link was distributed to participants via email and WhatsApp, capitalising on the broad reach and accessibility of these platforms [138,139]. To ensure data completeness and reduce missing responses, the survey required participants to answer all questions before submission, a practice known to improve data quality in online surveys [140]. The survey was designed to be completed within 10–15 minutes, which aligns with best practices for maintaining participant engagement and optimising response rates in digital surveys [141]. Before the primary data collection, a pilot test was conducted with 23 participants to identify and resolve any potential issues in the survey design. This pilot test was carried out before distributing the final survey to the target participants [142]. The individuals involved in the pilot test were excluded from the final analysis to maintain the study’s validity. A total of 123 complete responses were received for the final analysis, which was conducted using EFA and Confirmatory Factor Analysis to validate the tool.

## Qualitative analysis and discussion

This chapter presents an in-depth analysis and discussion of the qualitative data collected, shedding light on the key findings derived from the study. The chapter aims to highlight the emerging themes and patterns that provide a deeper understanding of the research questions by thoroughly examining the responses gathered through interviews and applying rigorous coding and thematic analysis techniques.

### Exploration of competitive dimensions

An in-depth analysis of the interview transcripts was conducted using NVivo for codification, identifying seven critical competitive dimensions within the apparel industry. These dimensions include customer satisfaction, innovation, facing change and uncertainty, better resource utilisation, collaboration to compete, strategic approach, and vertical advantage. Each dimension was organised into parent and child nodes, with parent nodes representing broader dimensions and child nodes acting as specific indicators of CP. The crosstab analysis, summarising the interview coding references, highlighted customer satisfaction (35.74%) and better resource utilisation (29.13%) as the most emphasised dimensions, signifying their importance in the apparel industry’s competitive landscape. These findings align with existing literature on the critical role of customer satisfaction and resource optimisation in maintaining competitive advantage (67, 68).

While customer satisfaction and resource utilisation emerged as dominant themes, other dimensions, such as collaboration to compete (15.62%), strategic approaches (8.71%), and innovation (6.91%), were moderately emphasised, reflecting their ongoing relevance in enhancing competitiveness. However, facing change, uncertainty, and vertical advantage was underrepresented, indicating potential gaps in strategic implementation. This underemphasis may expose adaptability and vertical integration vulnerabilities, which are crucial for navigating volatile markets and improving SC efficiencies [79]. The study’s findings suggest that focusing on customer satisfaction, resource utilisation, collaboration, innovation, and strategic orientation could serve as crucial areas for further exploration and performance measurement within the apparel SC, ultimately driving CP. Table 2 below shows the results of the heatmap of the crosstab query of CP dimensions.

**Table 2:** CP dimensions crosstab query results from the heatmap.

Nodes	INT - 1	INT - 2	INT - 3	INT - 4	INT - 5	INT - 6	INT - 7	INT - 8	All
Better Utilisation of Resources	38%	26.83%	23.40%	26.09%	42%	34.48%	18.18%	18.92%	29.13%
Collaborate to Compete	24%	14.63%	12.77%	23.91%	12%	10.34%	9.09%	13.51%	15.62%
Customer Satisfaction	24%	39.02%	36.17%	41.30%	14%	34.48%	57.58%	51.35%	35.74%
Facing the Change and Uncertainty	4%	0%	8.51%	0%	4%	3.45%	6.06%	0%	3.30%
Innovation	0%	9.76%	6.38%	6.52%	10%	10.34%	6.06%	8.11%	6.91%
Strategic Approach	10%	7.32%	10.64%	2.17%	18%	6.90%	3.03%	8.11%	8.71%
Vertical Advantage	0%	2.44%	2.13%	0%	0%	0%	0%	0%	0.60%

Five dimensions: customer satisfaction, efficient resource utilisation, collaboration for competitiveness, innovation, and strategic orientation consistently emerged across all interviews as critical to CP. These dimensions warrant further exploration to identify specific indicators that can effectively measure their contribution to CP.

### Exploration of digital capabilities

The qualitative analysis identified two major themes of DCs: process digitalisation (PD) and technical digitalisation (TD). These themes emerged as critical factors in understanding how digital tools and technologies are leveraged to enhance operational efficiency and drive organisational innovation. Both capabilities are pivotal in shaping an organisation’s digital transformation journey and are essential for achieving sustainable CP in today’s fast-paced, technology-driven environment.

#### Process digitalisation.

Process digitalisation is revolutionising industries by incorporating advanced technologies like flow synthesis, automation, analytics, and real-time control. These technologies drive data-driven discovery and production protocols, significantly improving process efficiency and sustainability across various sectors [143]. In this context, Business Process Management (BPM) plays a pivotal role in translating strategic goals into actionable execution. Digitalising Process Management (PoPM) fosters continuous improvement and agility. Integrating digital technologies ranging from big data and connectivity to analytics and automation has had profound impacts, particularly in chemical and biotechnological industries [144,145]. While digitalisation opens up numerous new business opportunities, it challenges companies to develop new ideas and products more agilely [146]. A comprehensive approach to digital process automation can optimise the entire value chain, especially in intralogistics, where process efficiencies can significantly impact overall performance. Despite these advancements, many process-driven industries have yet to fully harness the potential of digitalisation [147]. Table 3 below shows the key indicators identified under process digitalisation through the qualitative study.

**Table 3:** Process digitalisation dimensions crosstab query results from the heatmap.

Nodes	INT - 1	INT - 2	INT - 3	INT - 4	INT - 5	INT - 6	INT - 7	INT - 8	Total
AI and Prediction	37.50%	0%	15.38%	33.33%	25%	35.71%	50%	12.50%	28.57%
Advanced Analytics	62.50%	0%	38.46%	0%	16.67%	42.86%	0%	12.50%	24.68%
Customer and Supplier Portals	0%	100%	15.38%	25%	8.33%	7.14%	25%	25%	16.88%
Demand Planning Systems	0%	0%	0%	8.33%	8.33%	7.14%	0%	12.50%	5.19%
Inventory Management Systems	0%	0%	0%	0%	25%	7.14%	0%	25%	7.79%
Mechanical Automation	0%	0%	0%	8.33%	0%	0%	0%	0%	1.30%
Real-time Reliable Data	0%	0%	30.77%	25%	16.67%	0%	25%	12.50%	15.58%

#### Technical digitalisation.

Technical digitalisation has become a critical focus in the apparel industry, driven by the need to enhance efficiency, reduce lead times, and lower costs. Table 4 below presents the key indicators identified under technical digitalisation through a cross-tab analysis.

**Table 4:** Technical digitalisation dimensions crosstab query results from heatmap.

Nodes	INT - 1	INT - 2	INT - 3	INT - 4	INT - 5	INT - 6	INT - 7	INT - 8	Total
Digital Colour Management	0%	0%	0%	0%	60%	40%	50%	0%	25%
Digital Defects Detection	0%	0%	0%	0%	20%	0%	0%	0%	4.17%
Digital Fabric Inspection	0%	0%	0%	0%	20%	40%	0%	0%	12.50%
Digital Studio	0%	25%	0%	0%	0%	0%	0%	0%	8.33%
Virtual 3D Sampling	0%	75%	100%	100%	0%	20%	50%	100%	50%

One of the primary areas of focus is the automation of fabric inspection systems, which are increasingly adopted to improve quality control and reduce costs associated with manual inspection [148,149]. These systems utilise advanced technologies such as image processing, machine vision, and artificial intelligence to detect fabric defects more accurately and efficiently than human inspectors [150]. Automating the inspection process also helps reduce paper waste and streamline data management. Automated inspection systems can provide full fabric coverage, detect a wide range of defects, and significantly lower error margins than manual methods [151].

In addition to automated inspection, three-dimensional (3D) technologies are revolutionising apparel design and product development processes. Virtual prototyping allows designers to visualise garments digitally without needing physical samples, reducing time and costs [152,153]. 3D CAD systems are advancing rapidly, integrating body models, garment design, and fabric simulations to enable more interactive and precise product development [154]. These technologies foster faster decision-making, improved communication, and enhanced sustainability by minimising material waste and inefficiencies. 3D simulations, which consider fabric properties, body contact, and geometrical nonlinearity, provide more accurate representations of garment fit and appearance [155]. Although the adoption of 3D technologies has been slow, their perceived usefulness accelerates implementation within the industry [156]. Challenges remain, particularly in the need for physical samples to assess textile properties accurately [152]. Nevertheless, 3D virtual prototyping can potentially transform the global textile and apparel industry significantly, offering new opportunities for design, development, and marketing [157].

Moreover, digital colour management has become integral to the apparel and textile industry, evolving to cover various production processes. From early computer applications in the 1950s to advanced modern systems, digital colour management enables high-fidelity image communication and precise colour specifications [158]. The industry has seen significant advancements in digital printing technology, including software development, inkjet printing, and colour management systems that improve accuracy and consistency across the supply chain [159]. Digital tools for colour management encompass measurement, simulation, and quality control, ensuring that stringent colour tolerances are maintained throughout the textile manufacturing [160,161].

## Instrument development

The research team developed the tool following the recommended process outlined by Davis [162]. The process included the following steps: (1) identifying the tool concept through thorough qualitative research and a comprehensive literature review; (2) determining the formatting, writing, scoring, and comprehensibility of the tool; (3) conducting validity tests with an expert panel to assess the relevance and clarity of the questionnaire; (4) testing the reliability and validity of the data; and (5) performing factor analyses, including both CFA and EFA.

The identified indicators through the qualitative study were validated through an extensive literature review. Table 5 below presents the indicators identified through this qualitative research and subsequent literature review.

**Table 5:** Dimensions and indicators.

Dimensions	No	Indicators	Reference	Source
CSA: Customer Satisfaction	CSA1	Competitive unit price	[52,53]	Qualitative Study/ Literature
	CSA2	Consistently seek opportunities to optimise our pricing structure to serve customers better.	[52,53]	Qualitative Study/ Literature
	CSA3	Responsive to market dynamics and emerging opportunities.	[54,55]	Qualitative Study/ Literature
	CSA4	Agility to swiftly adapt to evolving market conditions	[54,55]	Qualitative Study/ Literature
	CSA5	Continuously improving production lead times.	[54]	Qualitative Study/ Literature
	CSA6	Delivering products promptly, outpacing competitors in speed of delivery.	[55–58]	Qualitative Study/ Literature
	CSA7	Information systems meticulously monitor all operational aspects.	[54]	Qualitative Study/ Literature
	CSA8	Real-time data tracking across supplies, finished goods, equipment, and personnel.	[54]	Qualitative Study/ Literature
	CSA9	Flexibility in adjusting product mix according to market demands.	[55,59]	Qualitative Study/ Literature
	CSA10	A diverse range of products to cater to varied customer preferences.	[53,60,61]	Qualitative Study/ Literature
	CSA11	Meet customer specifications for product features and technical requirements.	[62]	Qualitative Study/ Literature
	CSA12	Rigorous quality standards and processes to ensure product excellence.	[62]	Qualitative Study/ Literature
BUR: Better Utilisation of Resources	BUR1	Ongoing training to develop cutting-edge skills in the workforce		Qualitative Study
	BUR2	Continuous learning and development of organisational culture		Qualitative Study
	BUR3	Advanced forecasting tools to effectively manage and optimise resource capacity.		Qualitative Study
	BUR4	The ability to forecast shifts in the supply chain allows for agile resource reallocation.		Qualitative Study
	BUR5	Maintain minimal inventory levels of raw materials and finished goods to streamline operations.	[63,64]	Qualitative Study/ Literature
	BUR6	Production lines are finely tuned for optimal balance and efficiency.	[63,64]	Qualitative Study/ Literature
	BUR7	Low unit manufacturing costs compared to industry peers.	[55,57,59]	Qualitative Study/ Literature
	BUR8	Achieve high levels of on-time delivery performance.	[55,57,59]	Qualitative Study/ Literature
	BUR9	Investments in renewable energy sources to enhance resource sustainability.		Qualitative Study
	BUR10	Re-use and recycling methods to maximise resource utilisation efficiency.		Qualitative Study
CTC: Collaboration to Compete	CTC1	Share proprietary information and communicate events or changes (production, financial, design) that may impact supply chain partners.	[54,55,59]	Qualitative Study/ Literature
	CTC2	Supplier’s participation in new product development and design processes.	[54,55,59]	Qualitative Study/ Literature
	CTC3	Information exchange among suppliers, customers, and other external stakeholders to foster collaboration.	[54]	Qualitative Study/ Literature
	CTC4	Information sharing to facilitate collaborations with supply chain partners.	[55,65,66]	Qualitative Study/ Literature
	CTC5	Joint ventures or partnerships with supply chain actors to maximise strategic advantages		Qualitative Study
	CTC6	Data flows transparently between supply chain members, with full access for all firms to facilitate collaborative decision-making.	[54]	Qualitative Study/ Literature
	CTC7	Collaborative demand forecasting techniques with supply chain partners to enhance coordination and efficiency.	[54]	Qualitative Study/ Literature
STA: Strategic Advantage	STA1	Global geographical positioning provides a strategic advantage over competitors.		Qualitative Study
	STA2	High flexibility in adjusting manufacturing volume to meet market demands.	[55,59]	Qualitative Study/ Literature
	STA3	Leveraging trade agreements such as GSP, AGOA, etc., provides us with a strategic advantage.		Qualitative Study
	STA4	The visionary leadership		Qualitative Study
PD: Process Digitalisation	PD1	AI and advanced prediction technologies		Qualitative Study
	PD2	Advanced analytics to drive business decision-making processes.		Qualitative Study
	PD3	Utilises customer and supplier portals for seamless order placement, payments, and communication.		Qualitative Study
	PD4	Leveraging advanced demand planning technologies to plan sales and production capacities.		Qualitative Study
	PD5	Inventory management systems are used to optimise inventory and material handling.		Qualitative Study
	PD6	Automation across operations to streamline processes.		Qualitative Study
	PD7	Real-time data capture systems are integral to enhancing and expediting decision-making processes		Qualitative Study
TD: Technical Digitalisation	TD1	Advanced colour management technologies to precisely match thread, fabric shades, and other elements.		Qualitative Study
	TD2	Quality processes are enhanced with digital defect identification capabilities.		Qualitative Study
	TD3	Digital fabric inspection is integral to quality control measures.		Qualitative Study
	TD4	Digital studios to showcase products to customers effectively.		Qualitative Study
	TD5	Virtual 3D sampling capabilities in product development.		Qualitative Study

### Content validity

The research team engaged with 12 industry experts in Sri Lanka to validate the clarity and relevance of the instrument. Content validity was assessed using a Content Validity Index (CVI), where experts rated each item on a 4-point scale (1 = not relevant to 4 = highly appropriate). The degree of agreement on the relevance of the items was measured using the modified kappa coefficient, following the guidelines established by Polit, Beck [163]. Tilden, Nelson [164] and Gilbert and Prion [165] suggest that items with CVI values exceeding 0.70 are generally accepted; however Davis [166], recommends a CVI exceeding 0.80 as preferable. According to Perreault and Leigh [167], Kappa values ≤ 0 indicate no agreement, 0.01–0.20 indicate slight agreement, 0.21–0.40 indicate fair agreement, 0.41–0.60 indicate moderate agreement, 0.61–0.80 indicate substantial agreement, and 0.81–1.00 indicate almost perfect agreement. Consequently, this study eliminated items with CVI and Kappa values below 0.80 to ensure high content validity and agreement. Table 6 below shows the items that did not pass the threshold and were removed from the instrument.

**Table 6:** Items removed from the instrument at content validation.

Dimensions	Items Removed
CSA: Customer Satisfaction	CSA3, CSA4, CSA7, CSA8, CSA9
BUR: Better Utilisation of Resources	BUR1, BUR4, BUR8
CTC: Collaboration to Compete	CTC1

The developed tool comprised three main sections: participants’ demographic information, perceptions of the organisation’s CP, and perceptions of digital technological capabilities. The first section included items on organisation size, participants’ positions, and length of industry experience. These items were designed as a mix of open-ended and multiple-choice questions. The second and third sections comprised 36 items in total, addressing six key factors.

The Likert scale was selected for this study due to its widespread use in SEM applications [168]. There is ongoing debate among scholars regarding the ideal number of scale points and whether to use an even or odd number. According to Taherdoost [169], the validity and reliability of responses improve with a more extensive point scale, ideally between 7 and 11 points. Many studies incorporate a “neutral” midpoint in the Likert scale to account for respondents who may not have strong opinions. He, Van de Vijver [170] notes that cultural factors can influence how often individuals select middle response options.

Furthermore, people with lower socioeconomic status and education levels are more prone to acquiescence bias [171,172]. In Asian cultures, participants are likelier to choose the middle point or remain neutral, potentially leading to suboptimal results [173]. Considering this context and the study’s location in Sri Lanka, an 8-point Likert scale was utilised.

### Exploring the measurement scale: EFA

In this research, EFA was conducted using Varimax rotation, an orthogonal rotation strategy. Orthogonal rotations like Varimax are suitable when the primary goal is obtaining an interpretable and straightforward structure without assuming the factors are correlated. Varimax rotation maximises the variance of squared loadings of a factor matrix, simplifying interpretation by producing factors that are as distinct as possible [174]. Previous studies have demonstrated that Varimax rotation effectively clarifies factor structures, making it a widely accepted method in various fields. The selection of orthogonal rotation (Varimax) was guided by both empirical results and the objective of achieving a simplified and interpretable factor structure. Although digital capabilities and competitive performance are theoretically interlinked, an empirical test using oblique rotation (Promax) revealed relatively low inter-factor correlations (below 0.3), suggesting that the latent constructs are sufficiently distinct in the context of this study. According to Hair et al. [129], orthogonal rotation is suitable when factor correlations are low, as it produces uncorrelated factors that are easier to interpret and compare across studies [129]. Additionally, orthogonal rotation helps reduce multicollinearity among factors, which is beneficial when proceeding to subsequent analyses like CFA or SEM [175]. Given the objective of developing a validated and practically applicable measurement instrument, Varimax rotation allowed us to extract clean factor loadings with minimal cross-loadings, enhancing the clarity and utility of the resulting constructs.

The factor loading criteria were set at 0.50, ensuring that each item significantly contributes to its respective factor [176]. Additionally, the commonality of the scale, indicating the amount of variance explained by each factor, was assessed to ensure acceptable levels of explanation. The results showed that all communalities exceeded 0.50, indicating a solid reason for variance. Bartlett’s Test of Sphericity was employed to determine the overall significance of the correlation matrix, providing a measure of the statistical probability that significant correlations exist among the matrix components. The results were substantial, χ2 (n = 123) = 6084.675 (p < 0.000), indicating the suitability of the data for factor analysis [177,178]. Additionally, the Kaiser–Meyer–Olkin (MSA) measure of sampling adequacy was calculated, yielding a value of 0.853. Data with MSA values above 0.800 are highly suitable for factor analysis [178]. The criteria for item deletion in this research were based on established guidelines in the literature. According to Costello and Osborne [179], high factor loadings greater than 0.70 indicate strong relationships between items and their underlying factors, thereby enhancing the reliability and interpretability of the scale. Furthermore, cross-loading items can obscure factor definitions and lead to ambiguous results, justifying their removal when cross-loadings exceed 0.32 [180]. Therefore, items with loadings less than 0.70 were removed to ensure each item significantly contributed to its respective factor. Additionally, items with cross-loadings greater than 0.32 on at least two factors were considered for deletion, mainly if other items had factor loadings of 0.50 or higher. This approach aligns with recommendations to enhance the clarity and distinctiveness of the factors [174,179].

The factor solution derived from this analysis yielded six factors, accounting for 83.104 per cent of the variance in the data. However, in this initial EFA, two items failed to meet the criteria for retention. Specifically, item PD1: “We harness AI and advanced prediction technologies to inform our strategies” did not load significantly on any dimension, and item PD2: “Advanced analytics drive our business decision-making processes” loaded onto a factor other than its intended one. Consequently, these two items were removed from further analysis to ensure the integrity and validity of the factor structure.

The authors conducted the EFA again, excluding the previously identified items. The results of this revised analysis confirmed the six-dimensional structure as theoretically defined in the research. The results in Table 7 revealed six factors explaining a significant proportion of the variance in the data.

**Table 7:** EFA total variance explained.

Component	Initial Eigenvalues % of the variance	Cumulative %	Rotation Sums of Squared Loadings % of variance	Cumulative %
1	26.568	26.568	17.699	17.699
2	22.563	49.131	15.336	33.035
3	12.752	61.882	14.842	47.877
4	9.720	71.602	13.229	61.106
5	6.841	78.443	12.100	73.206
6	5.643	84.086	10.880	84.086

The initial eigenvalues indicated that these six factors accounted for 83.104% of the total variance. Specifically, Factor 1 explained 28.647% of the variance, Factor 2 explained 21.329%, Factor 3 explained 12.043%, Factor 4 explained 9.220%, Factor 5 explained 6.465%, and Factor 6 explained 5.400%. The cumulative variance explained by these six factors after rotation was 83.104%, demonstrating a robust factor structure [174,176].

Bartlett’s Test of Sphericity was significant, χ^2^ (n = 123) = 5693.700 (p < 0.000), confirming the presence of adequate correlations among the items, and all communalities exceeded the required value of 0.500, ensuring robust explanatory power. Table 8 below shows the rotated component matrix.

**Table 8:** Rotated Component Matrix loadings.

Items	Component					
	1	2	3	4	5	6
CSA1	0.933					
CSA2	0.929					
CSA5	0.920					
CSA6	0.851					
CSA10	0.922					
CSA11	0.897					
CSA12	0.897					
BUR2		0.750				
BUR3		0.886				
BUR5		0.864				
BUR6		0.857				
BUR7		0.768				
BUR9		0.865				
BUR10		0.855				
CTC2			0.883			
CTC3			0.899			
CTC4			0.888			
CTC5			0.858			
CTC6			0.800			
CTC7			0.766			
STA1						0.893
STA2						0.895
STA3						0.892
STA4						0.899
PD3				0.930		
PD4				0.925		
PD5				0.915		
PD6				0.926		
PD7				0.759		
TD1					0.924	
TD2					0.883	
TD3					0.822	
TD4					0.925	
TD5					0.887	

Extraction Method: Principal Component Analysis. Rotation Method: Varimax with Kaiser Normalisation.

**Fig 2:**
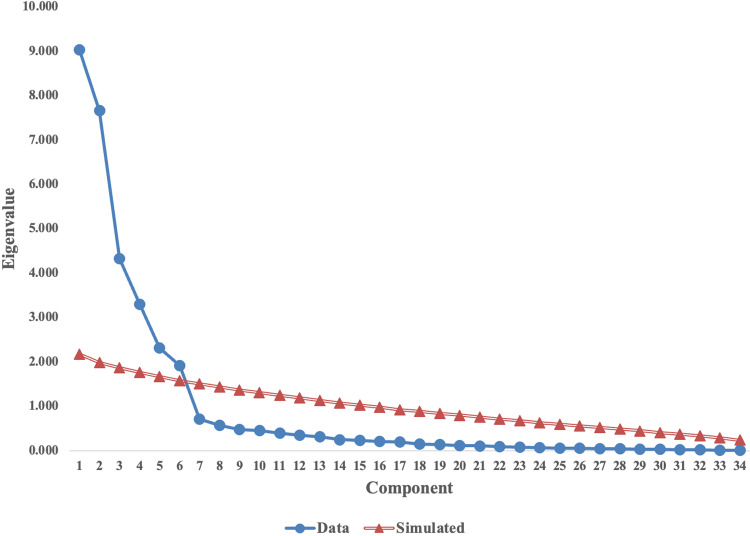
Parallel analysis scree plot.

Relying solely on the eigenvalue > 1 rule is insufficient, as it has been demonstrated to be one of the least accurate methods for assessing factor retention [179]. Therefore, parallel analysis was conducted to provide a more robust evaluation. For an item to be retained, the acceptable eigenvalue must be greater than one [181,182]. Patil, Surendra [183] was utilised to calculate the eigenvalues from randomly generated correlation matrices. These random eigenvalues were then compared with those extracted from the research dataset. The number of factors to retain corresponds to the number of eigenvalues from the researcher’s dataset that exceed the random eigenvalues [184]. The scree plot in Fig 2 indicated six factors to retain.

The six factors identified through the EFA aligned with the theoretical propositions of the research. Factor 1 represents Customer Satisfaction (CSA). Factor 2 represents Better Utilisation of Resources. Factor 3 pertains to Collaboration to Compete (CTC). Factor 4 represents Strategic Advantage (STA). Factor 5 pertains to Process Digitalisation (PD). Finally, Factor 6 represents Technical Digitalisation.

### Confirmatory factor analysis

Researchers and reviewers must independently evaluate the results obtained in an EFA study. This should occur on two levels: first, independent researchers should assess the analytic choices made by the authors, considering the many subjective decisions involved in EFA. Second, they should be able to replicate the study accurately using new data or conduct a CFA to verify the findings [185]. Therefore, in this study, CFA was employed to ensure the robustness of the findings.

#### Normality and reliability of data.

First, the researchers tested the normality of the data and internal consistency using SPSS software. According to Kline [130], absolute skewness values greater than 3.0 indicate an extremely skewed distribution, absolute kurtosis values greater than 10.0 suggest a problem, and values exceeding 20.0 indicate a potentially serious issue with Kurtosis. As shown in Table 9, the skewness and kurtosis values of the dataset were well within these acceptable ranges, indicating that the sample data is normally distributed.

**Table 9:** Skewness, Kurtosis, and Cronbach’s alpha values.

Items by Subscale	Max	Min	Mean	Skewness	Kurtosis	Cronbach’s Alpha
Customer Satisfaction						0.971
CSA1	4	8	7.19	−1.063	−0.305	
CSA2	4	8	7.13	−1.063	−0.294	
CSA5	4	8	7.11	−1.114	−0.036	
CSA6	4	8	6.89	−0.652	−0.629	
CSA10	4	8	6.88	−0.778	−0.375	
CSA11	4	8	6.74	−0.594	−0.455	
CSA12	4	8	6.82	−0.681	−0.363	
Better Utilisation of Resources						0.937
BUR2	2	7	5.28	−1.61	2.936	
BUR3	3	7	5.98	−0.786	−0.38	
BUR5	4	7	5.85	−0.333	−1.022	
BUR6	3	7	5.89	−0.569	−0.331	
BUR7	2	6	5.04	−0.835	0.25	
BUR9	2	7	5.98	−0.787	0.716	
BUR10	1	6	4.39	−1.507	2.897	
Collaborate to Compete						0.949
CTC2	3	7	5.07	−0.555	−0.631	
CTC3	2	7	4.36	0.009	−1.012	
CTC4	1	6	3.39	0.085	−0.999	
CTC5	2	5	3.92	−0.84	−0.532	
CTC6	1	5	2.5	0.025	−1.247	
CTC7	1	4	2.38	−0.005	−1.24	
Strategic Advantage						0.979
STA1	2	6	4.8	−0.372	−0.803	
STA2	1	5	3.78	−0.364	−0.759	
STA3	3	7	5.76	−0.83	0.197	
STA4	3	7	5.4	−0.259	−1.331	
Process Digitalisation						0.966
PD3	2	5	3.87	−0.456	−0.336	
PD4	2	5	3.85	−0.405	−0.438	
PD5	4	7	5.73	−0.439	−0.008	
PD6	3	6	4.79	−0.469	−0.036	
PD7	2	7	3.86	0.239	0.123	
Technical Digitalisation						0.932
TD1	1	5	2.8	0.085	0.114	
TD2	1	4	1.93	0.603	−0.102	
TD3	1	3	2.07	0.154	1.011	
TD4	3	6	4.63	−0.464	−0.386	
TD5	3	6	4.6	−0.27	−0.78	

Cronbach’s alpha was calculated to assess the scale’s internal consistency and reliability. For the 36-item full scale, Cronbach’s alpha was 0.903, demonstrating excellent internal consistency [186]. Similarly, each subscale showed alpha values greater than 0.90, supporting the instrument’s reliability. These findings confirm that the data are typically distributed and internally consistent, making them suitable for further analysis.

#### First-order model CFA results.

**Fig 3:**
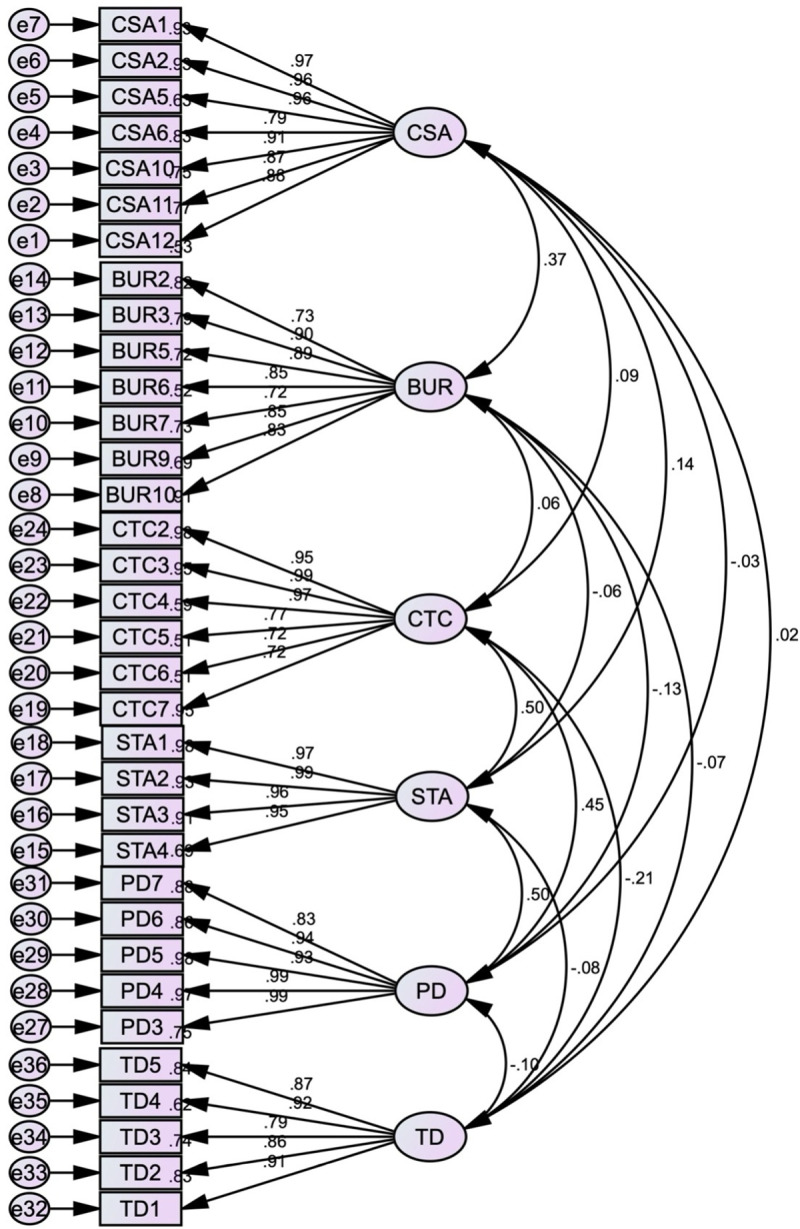
First-order initial CFA model.

The CFA was conducted to assess the validity of the measurement model. The results, including factor loadings and model fit indices, provide insights into the model’s adequacy and areas for improvement. The standardised estimates produced by Amos, using Maximum Likelihood (ML) estimation to handle missing data, are presented in Fig 3.

The factor loadings for the observed variables on their respective latent constructs are robust, ranging from 0.59 to 0.99. These high factor loadings suggest that the observed variables are strong indicators of their underlying constructs, contributing positively to the overall measurement model. Generally, factor loadings above 0.70 are considered very good, while loadings above 0.50 are acceptable for exploratory models [187]. In this analysis, most factor loadings exceed 0.70, affirming the reliability and validity of the constructs.

The overall model fit was evaluated using several indices, providing a comprehensive assessment of the model’s suitability. CMIN/DF was 2.082 (p < 0.05), which falls within the acceptable range of 2–5, indicating a reasonable fit of the model to the data [188]. This suggests that the model appropriately represents the observed data, though the ratio alone should not be relied upon without considering other fit indices. GFI and AGFI were 0.669 and 0.615, respectively. These values are below the recommended threshold of 0.90, indicating that the model could be improved [189]. The relatively low GFI and AGFI values suggest that the model does not achieve an optimal fit, possibly due to model complexity or sample size. The NFI, IFI, TLI, and CFI were 0.831, 0.905, 0.895, and 0.902, respectively. These indices are close to the acceptable threshold of 0.90, indicating that the model reasonably explains the variance and covariance among the variables [190]. Although not ideal, these values suggest that the model moderately fits the data. RMSEA was 0.094, with a 90% confidence interval ranging from 0.086 to 0.102. This value exceeds the generally accepted threshold of 0.08, suggesting that the model has some degree of misfit [191]. A higher RMSEA value indicates that the model could be further refined to fit the data better. The AIC for the default model was 1231.908, significantly lower than that of the independence model (6392.413), suggesting that the hypothesised model is more appropriate [192]. The ECVI for the default model was 10.098, much lower than that of the independence model (52.397), indicating that the model is likely to perform better with new data. Hoelter’s Critical N for the default model was 65 at the 0.05 significance level, indicating that the sample size is marginally sufficient for detecting the model’s structure [193]. A larger sample size might provide more stable and reliable estimates.

Due to the initial model’s suboptimal fit, the researchers considered modifications based on modification indices (MI) and the examination of residuals. This approach is commonly recommended in structural equation modelling (SEM) when the initial model fit is inadequate [189]. The model was iteratively tested by removing eight items, CSA6, BUR2, BUR10, CTC6, CTC7, PD1, PD2, and PD7, which exhibited high standardised residual values that exceeded acceptable thresholds [194].

Additionally, covariances were added between specific error terms where high MI suggested that doing so would improve model fit. Specifically, covariances were added between the following pairs of error terms: e1 - e2, e1 - e3, e2 - e3, e9 - e10, e21 - e24, e29 - e30, e32 - e33.

**Fig 4:**
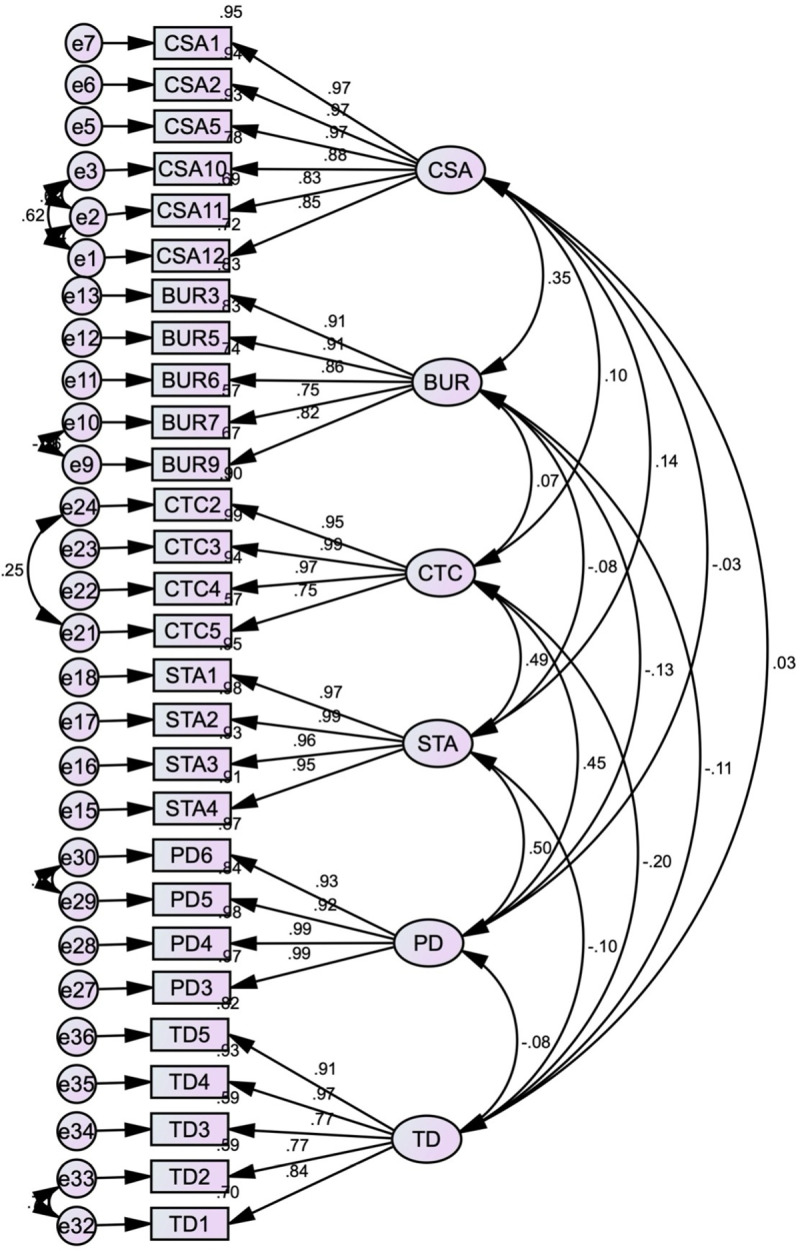
First order final model.

The modified model, as shown in the updated CFA diagram and the corresponding model fit indicators, demonstrates significant improvement in fit compared to the initial model. Fig 4 below presents the standardised estimates of the adjusted first-order model, calculated using ML estimation, along with the updated model fit indices, which show an improved fit after these modifications.

The standardised estimates for the factor loadings in the modified model remain strong, with values ranging from 0.62 to 0.99. These high loadings suggest that the observed variables are strong indicators of their respective latent constructs, contributing positively to the overall measurement model. Removing the previously problematic items has likely contributed to a more stable and reliable factor structure.

The CMIN/DF ratio for the modified model is 1.183 (p < 0.05), which is well within the acceptable range of 1–3, indicating an excellent fit between the model and the observed data. This is a significant improvement from the initial model, suggesting that the modifications made to the model were effective. RMR is 0.037, which is low, indicating a good fit. GFI is 0.827, and the AGFI is 0.786. While GFI and AGFI are slightly below the commonly accepted threshold of 0.90, they still indicate a reasonably good fit, considering the complexity of the model and the sample size. NFI is 0.927, the IFI is 0.988, the TLI is 0.986, and the CFI is 0.988. These indices are above the recommended threshold of 0.90, indicating that the model fits the data exceptionally well. These high values further confirm the effectiveness of the modifications. RMSEA is 0.039, with a 90% confidence interval ranging from 0.019 to 0.053. An RMSEA below 0.05 indicates a close fit of the model with the degrees of freedom.

Additionally, the PCLOSE value of 0.893 suggests that the probability of the RMSEA being below 0.05 is high, further supporting the model’s adequacy. The AIC for the modified model is 544.135, significantly lower than that of the independence model (5362.23), indicating that the modified model is far superior in explaining the data. The ECVI is 4.46, which is lower than that of both the saturated and independent models, suggesting that the modified model would better predict new data. Hoelter’s Critical N is 117 at the 0.05 significance level, indicating that the sample size is adequate for detecting the model’s structure. This value is much higher than the critical N of the independence model, further affirming the adequacy of the modified model.

#### Second-order model CFA results.

**Fig 5:**
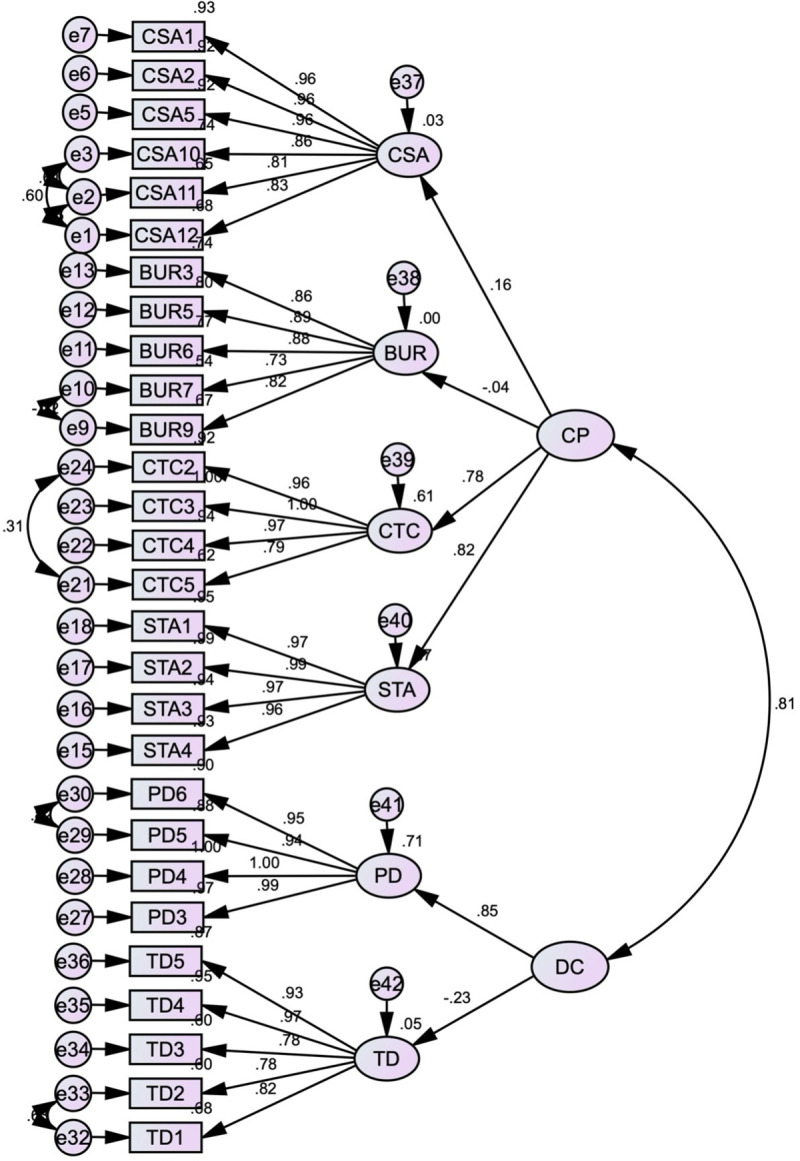
Second-order final model.

This study employed a second-order CFA model to assess the relationships among multiple latent constructs. The Generalised Least Squares (GLS) method was chosen for parameter estimation due to its superior ability to handle the complexities inherent in second-order models, such as heteroscedasticity, correlated errors, and potential deviations from multivariate normality [130,191,195]. Fig 5 below presents the standardised estimates of the adjusted first-order model, calculated using GLS estimation, along with the updated model fit indices, which show an improved fit after these modifications.

In the second-order CFA model, the first-order latent constructs (CSA, BUR, CTC, STA, PD, and TD) load onto higher-order constructs (CP and DC). The factor loadings for the observed variables on their respective first-order constructs remain strong, ranging from 0.60 to 1.00. This indicates that the observed variables are well represented by their respective latent constructs. Additionally, the second-order loadings show a strong relationship between the first-order constructs and the overarching factors CP and DC. For instance, PD and TD show strong loadings onto DC, underscoring the importance of digital processes and technical digitalisation in the broader construct of DCs.

The CMIN/DF ratio for the default model is 0.989 (p > 0.05), which is below 1, indicating an excellent fit. A CMIN/DF value close to or below two is typically considered a sign of a well-fitting model. RMR is 0.189, which suggests a reasonably good fit, though not ideal. The GFI is 0.806, and the AGFI is 0.765, both of which are slightly below the recommended threshold of 0.90 but still indicate a reasonable fit considering the complexity of the model. The NFI is 0.499, below the recommended threshold of 0.90, indicating a moderate fit. However, the IFI, TLI, and CFI are around 1.0 (IFI = 1.012, TLI = 1.015, CFI = 1.0), indicating a perfect fit. These high values suggest that the model fits the data well compared to a baseline model. The RMSEA for the default model is 0, with a 90% confidence interval ranging from 0 to 0.032. This exceptionally low RMSEA indicates a close fit between the model and the observed data. Additionally, the PCLOSE value of 1 indicates a high probability that the RMSEA is below 0.05, further supporting the model’s adequacy. The AIC for the default model is 472.173, significantly lower than that of the independence model (718.492), indicating that the default model is better suited for explaining the data. The ECVI is 3.87, lower than the saturated and independent models, suggesting that the default model would better predict new data. Hoelter’s Critical N is 140 at the 0.05 significance level, indicating that the sample size is adequate for detecting the model’s structure. This value is much higher than the critical N of the independence model, further affirming the adequacy of the modified model. The overall fit indices strongly support the validity and reliability of the model. The substantial factor loadings and well-fitting higher-order constructs confirm the theoretical underpinnings of the model, providing a solid basis for further research and application.

### Determine the best model

When using SEM, constructing a single model is the most straightforward approach. However, it is often insufficient to analyse only one model. Instead, it is more appropriate to assess multiple competing models and compare the results to understand better and identify the best-fitting model for the data [196]. Different models can be compared regarding their fit by performing a chi-square (χ²) difference test. This test enables researchers to determine whether one model fits the data significantly better or worse than a competing model. Whether both models are equivalent can be tested using the following hypothesis.


$$H_{0}:\;\Delta \chi^{2}\Delta df=0$$

$$H_{1}:\;\Delta \chi^{2}\Delta df>0$$

Below are the formulas to obtain $\Delta \chi^{2}$ and Δdf between the first and second-order models.


$$\Delta \chi^{2}=\;\chi^{2}(second\;order\;model)-\;\chi^{2}(first\;order\;model)=\;4.96$$

Δdf= df(second order model)− df(first order model) = 8

Alpha was calculated using an Excel formula, and the P-value is 0.756. The result was not significant at p < 0.05. This means H_1_ was rejected and accepted H_0._ That $\Delta \chi^{2}$ there is no significant difference between first and second-order models.

## Discussion

The conclusion of this study integrates the findings from the in-depth qualitative analysis and the quantitative validations to underscore the critical role of DCs and CP within the apparel industry.

The first two objectives of this study are to explore DCs and CP dimensions within the apparel industry. This study identified six empirically validated dimensions, four representing CP and two representing DC, that offer a holistic framework to understand digital transformation and strategic competitiveness in the apparel supply chain. Each dimension reflects a distinct yet interconnected performance or capability domain critical in today’s volatile, digitally driven market environment.

Customer Satisfaction emerged as a fundamental dimension of competitive performance, reinforcing its long-established role as both an outcome and driver of supply chain excellence. In the context of the apparel industry, where market responsiveness and short product life cycles are the norm, customer satisfaction reflects the ability to deliver quality, speed, customisation, and service reliability [197]. This dimension aligns with prior research emphasising the role of customer-centric strategies in enhancing brand loyalty and market positioning. It also confirms that digital capabilities contribute to satisfaction by enabling better demand forecasting, product personalisation, and omnichannel fulfilment [198]. Better Utilisation of Resources reflects the operational efficiency aspect of performance, focusing on how effectively an organisation allocates and manages its physical, human, and technological resources. Efficient resource utilisation is a key competitive lever in the apparel sector, often constrained by cost pressures and labour intensity. This dimension is closely associated with lean manufacturing principles and sustainable operations. The findings support previous research that links digital process integration with reductions in waste, idle time, and cost overruns [199], suggesting that digitalisation enhances speed and precision in resource deployment. This dimension reflects the shift from firm-centric competition to network-based competitiveness, where collaboration across supply chain partners becomes essential. The emergence of Collaboration to Compete aligns with literature on supply chain integration and relational capabilities [200,201]. In an industry where coordination with fabric suppliers, logistics providers, and buyers is vital, collaboration enabled by digital platforms (e.g., shared PLM systems, cloud-based dashboards) is a key performance enabler. This dimension also ties into the ERBV, highlighting the strategic importance of leveraging inter-organisational resources and partnerships [202]. Strategic Advantage captures a firm’s ability to leverage digital capabilities to create differentiation and long-term market positioning. Unlike short-term operational gains, this dimension reflects higher-order outcomes such as innovation, resilience, market leadership, and brand equity. It connects with the DCV, emphasising firms’ need to sense opportunities, seize them through resource mobilisation, and transform accordingly [36]. In a hyper-competitive apparel market, achieving strategic advantage requires going beyond efficiency to embrace continuous innovation, fast fashion responsiveness, and data-driven strategic decision-making.

Technical Digitalisation refers to the foundational IT infrastructure and technological readiness that support digital transformation. This includes investment in hardware, software, cloud platforms, data analytics tools, and cybersecurity systems. It reflects a firm’s capacity to acquire and maintain the technical resources necessary for digital operations. Previous studies suggest that technical IT capability is a necessary but insufficient condition for digital transformation; it lays the groundwork but must be complemented by process and strategic alignment [203,204]. In this study, TD is thus positioned as a base layer that enables higher-level capabilities and outcomes. In contrast, Process digitalisation reflects how digital tools are embedded into day-to-day business and operational processes. This includes using digital technologies in production scheduling, inventory management, quality control, and customer service workflows. PD is a dynamic capability that enables process-level agility, visibility, and automation. It operationalises the transformation of workflows through technology and is essential in bridging the gap between IT investment and actual performance outcomes [205,206]. Importantly, PD supports internal process excellence and external responsiveness, making it critical to achieving both CSA and STA.

Together, these six dimensions provide a multidimensional framework that captures the means (digital capabilities) and ends (competitive performance) of digital transformation in apparel supply chains. Their validation through empirical analysis strengthens their theoretical robustness and practical utility. They respond to scholarly calls for models that move beyond narrow operational metrics to include strategic, relational, and digital elements [207,208]. Moreover, the distinction between TD and PD allows for more nuanced diagnostics and capability development strategies, particularly in resource-constrained environments like Sri Lanka.

The third objective of this study was to develop a robust research instrument that accurately measures the constructs relevant to CP and DCs in apparel manufacturers through a rigorous process involving EFA and CFA. The study successfully identified and validated six key factors that should be included in the final questionnaire. These factors are critical for capturing the underlying dimensions of CP and DCs.

The six validated factors that should be incorporated into the research instrument. CSA, which measures an organisation’s effectiveness in meeting customer expectations and delivering value, is a crucial driver of CP. BUR captures how efficiently an organisation manages and optimises its resources, contributing to operational excellence and competitiveness. CTC assesses how organisations collaborate internally and externally to enhance their competitive edge in the marketplace. STA includes an organisation’s ability to leverage its strengths to gain and maintain a strategic advantage over competitors. PD focuses on implementing digital technologies to improve organisational processes essential for achieving operational efficiency and innovation. TD measures the extent to which an organisation adopts and integrates technical DCs to enhance its overall performance and competitiveness.

The final questionnaire should include items that comprehensively measure these six factors. Each factor has been shown to have solid loadings and clear contributions to the overarching constructs of CP and DCs. By including these validated factors, the research instrument will provide a reliable and valid tool for assessing the impact of various organisational strategies on performance outcomes.

In the first-order CFA model, the standardised factor loadings were robust, ranging from 0.62 to 0.99, confirming the reliability and validity of the constructs. While the initial model did not fit optimally, iterative adjustments based on modification indices improved the model fit significantly, as evidenced by fit indices such as CMIN/DF (1.183), RMR (0.037), and CFI (0.988). These results indicate that the modified first-order model provided an excellent fit for the data. The second-order CFA model refined the analysis by grouping the first-order latent constructs into higher-order constructs, demonstrating strong relationships between first-order constructs and overarching factors such as CP and DC. The model fit indices, including a CMIN/DF ratio of 0.989 and RMSEA of 0, suggested an exceptional model fit. The model’s strong factor loadings and fit indices provide robust support for the theoretical structure and practical application of the model.

When comparing the first-order and second-order models, the chi-square difference test revealed no significant difference between the two models, suggesting that both models fit the data similarly well. This indicates that the simpler first-order model may be sufficient for capturing the relationships between constructs. Still, the second-order model offers additional theoretical depth by consolidating related factors into higher-order constructs. Depending on the research objectives, either model could be applied effectively.

### Theoretical contribution

This chapter articulates how the study offers novel theoretical insights and builds upon existing frameworks to deepen the academic discourse.

#### Introduction of a new measurement instrument.

This study makes a substantive theoretical advancement by introducing and empirically validating a multidimensional measurement instrument that captures the complex interplay between DC and CP within the apparel supply chain context. Existing literature on digital transformation in supply chains has been hindered by fragmented constructs, inconsistent operational definitions, and a lack of standardised tools, which collectively undermine theoretical coherence and limit cross-study comparability [203,209]. This research addresses a critical gap by developing a conceptually integrated and empirically robust instrument. It offers a foundation upon which future theoretical work in digital transformation and supply chain competitiveness can be rigorously built.

This study moves beyond sector-specific or technology-centric perspectives that often narrowly define digital capabilities. Instead, it reconceptualises DC through two interrelated dimensions, TD and PD, thereby capturing infrastructural readiness and process-level digital integration. This bifocal approach aligns with the DCV by illustrating how firms acquire digital technologies and embed them into operational routines to sense, seize, and transform in dynamic markets. Furthermore, the instrument identifies four interdependent CP dimensions, CSA, BUR, CTC, and STA, representing a nuanced understanding of competitive performance beyond traditional metrics such as cost or speed. These dimensions provide empirical grounding to theoretical constructs such as supply chain agility, value co-creation, and strategic alignment [197,210]. This contribution is not merely descriptive; it is diagnostic and explanatory. For academics, the instrument offers a validated, theory-driven tool for systematically examining how digitalisation translates into measurable performance outcomes. It facilitates longitudinal analysis and hypothesis-driven research, enabling scholars to explore causal pathways and contingencies in digital transformation efforts. The rigorous application of EFA and CFA enhances construct validity and reliability, making the instrument suitable for replication and comparative analysis across sectors and geographies.

Additionally, this framework advances theoretical dialogue by enabling investigations into digital capabilities’ mediating or moderating roles in the resource–performance link, as posited in the RBV and ERBV. It also creates new avenues for cross-national research to explore contextual enablers and constraints of digital transformation, particularly in emerging economies where digital maturity and institutional dynamics differ. This instrument is not merely a measurement tool but a conceptual innovation that integrates digital capabilities with multifaceted performance outcomes in supply chains. It directly addresses long-standing critiques in the literature regarding the lack of empirical clarity and actionable insights in digital transformation studies [207]. It lays the groundwork for a more mature, empirically grounded theoretical discourse in the digital supply chain domain.

#### RBV, DCV, and ERBV alignment.

This study advances theoretical discourse by developing and empirically validating a multidimensional measurement instrument for assessing DC and CP within the apparel supply chain, grounded in the RBV, DCV, and ERBV These complementary theoretical lenses offer a robust foundation to conceptualize digital capabilities not merely as technical enablers but as strategically embedded, dynamic resources that drive sustainable competitive advantage in volatile, digitized environments.

From the RBV perspective, this study operationalises TD and PD as valuable, rare, inimitable, and non-substitutable resources (VRIN). These digital capabilities directly contribute to firm-level outcomes such as CSA and BUR, reinforcing the argument that digitisation is not merely infrastructural but a strategic asset embedded within organisational routines. The differentiation between TD and PD enables a more nuanced understanding of how heterogeneous digital assets contribute to internal efficiency and external responsiveness, core tenets of sustained performance in the RBV framework. Building on the DCV, the study further demonstrates how digital capabilities enable firms to sense, seize, and transform in response to environmental dynamism. As dimensions of CP, the inclusion of STA and CTC highlights how digital tools empower firms to reconfigure their resource base and align internal and external competencies to capitalise on emerging opportunities. The validated measurement model, therefore, provides empirical grounding for the claim that dynamic capabilities, activated through digitalisation, facilitate agility, innovation, and long-term strategic positioning in the supply chain ecosystem. Moreover, the ERBV is explicitly addressed by incorporating externally embedded digital resources. The study acknowledges that collaborative platforms, cloud-based ecosystems, and digital interfaces with suppliers and customers represent inter-organisational assets. While not owned, these resources are co-created and accessed through digital interconnectivity, playing a pivotal role in enabling CTC and amplifying competitive performance beyond firm boundaries. This aligns with recent calls in the literature to expand RBV-based theorising to account for relational and ecosystem-level digital assets that contribute to sustainable value creation.

This study transcends traditional firm-centric views by bridging RBV, DCV, and ERBV and positions digital transformation as a multi-level, capability-driven phenomenon. The validated measurement instrument provides a theoretically coherent and empirically rigorous foundation for future studies to explore how internal digital capabilities and external partnerships collectively shape competitive trajectories in digitally intensive sectors. In doing so, it offers a significant leap toward integrated theorisation of digital transformation in supply chains grounded in empirical precision and aligned with evolving theoretical paradigms.

### Practical contribution

This study contributes to apparel supply chain practice by introducing a rigorously validated, multidimensional measurement instrument that enables organisations to assess and strategically manage their digital transformation efforts. Far beyond a theoretical construct, this instrument provides a practical, decision-support framework that helps firms translate digital capability assessments into actionable insights for performance improvement.

By using this tool, organisations can systematically evaluate their digital maturity across key dimensions, specifically, PD and TD, and directly link these capabilities to critical competitive performance outcomes such as agility, cost-efficiency, responsiveness, innovation, and customer satisfaction. This enables firms to identify existing capability gaps, diagnose underperforming areas, and prioritise digital investments that yield the greatest strategic return. Importantly, the instrument is flexible and scalable, making it equally relevant for small and medium-sized enterprises (SMEs) and larger firms. SMEs, often limited by financial and human capital, can use the tool to focus on incremental, high-impact digital solutions such as cloud-based analytics, workflow automation, or e-procurement platforms. Meanwhile, larger organisations can leverage the framework to guide the integration of advanced technologies like AI, IoT, and predictive analytics into complex, multi-tiered supply chain operations.

In practice, this tool supports a wide range of strategic applications:

- Benchmarking digital readiness against industry peers or internal standards.
- Aligning digital initiatives with firm-specific capabilities and strategic objectives.
- Guiding resource allocation toward the most impactful digital interventions.
- Monitoring progress of digital transformation initiatives over time.
- Supporting change management and capability development across functional areas.

The measurement instrument bridges the persistent gap between conceptual understanding and practical execution. It empowers apparel supply chain leaders to move from ad hoc, intuition-based digital adoption toward a structured, evidence-based transformation approach. It enables organisations to navigate technological disruption with greater confidence, resilience, and competitiveness in today’s dynamic global apparel landscape.

Although this study was conducted within the Sri Lankan apparel manufacturing context, the validated measurement instrument has strong potential for adaptation and application in other apparel-producing countries, particularly those with similar industry structures and developmental profiles. Emerging economies such as Bangladesh, Vietnam, India, and Ethiopia share comparable challenges, including cost competitiveness, lean margins, export dependency, and increasing pressure to digitise operations. These countries operate within global value chains and are transitioning similarly toward process automation, collaborative platforms, and digital supply chain visibility [211]. While contextual factors such as regulatory frameworks, digital infrastructure, and workforce capabilities may influence the speed and scope of adoption, the instrument provides a structured, diagnostic foundation that can be localised and validated to suit different national settings. This opens pathways for comparative studies and benchmarking initiatives across regions, promoting shared learning and strategic alignment in the global apparel supply chain.

### Limitations and future research directions

Despite its contributions, this study is not without limitations, which should be acknowledged to contextualise the findings and guide future research. First, the research was geographically confined to apparel manufacturers operating in Sri Lanka. While this focus provided in-depth insights into the dynamics of DCs and CP within a key emerging market, the findings may not be fully generalizable to other countries or industries with different institutional, cultural, and technological contexts. The specific characteristics of the Sri Lankan apparel sector, including infrastructure maturity, regulatory environment, and digital readiness, may limit the applicability of the results to broader or more advanced manufacturing contexts. Second, the study employed a cross-sectional survey design and a relatively small sample size, which restricts the ability to infer causal relationships and limits the robustness of generalisations. A longitudinal approach would be better suited to capture the evolution of digital transformation efforts and their sustained impact on competitive performance over time. Third, using an 8-point Likert scale in data collection to reduce neutrality may have introduced response bias, particularly within the Asian cultural context. Research has shown that respondents in collectivist societies often exhibit moderate response tendencies or social desirability bias, potentially skewing self-reported measures [171,212]. This cultural response pattern could affect the precision of constructs like perceived digital capability and performance outcomes, and future studies may consider triangulating with objective data sources to mitigate this limitation.

Future research could address these constraints by expanding the study to include apparel manufacturers in other countries and across different regions, particularly South Asia and Southeast Asia, to assess the external validity of the proposed framework. Comparative studies could illuminate regional variations in digital capability adoption and performance outcomes, enabling a more comprehensive understanding of context-specific enablers and barriers.

Moreover, future studies should broaden the scope to incorporate key supply chain actors such as suppliers, logistics providers, and retailers to capture the collaborative dimension of digital transformation. Exploring how end-to-end digitisation and partner integration impact CP would offer a more holistic view of value chain competitiveness. Additionally, examining the role of emerging technologies like blockchain, IoT, and artificial intelligence in shaping digital maturity and inter-firm collaboration presents a promising avenue for future inquiry.

A mixed-methods or longitudinal design could enrich future research by combining self-reported perceptions with objective performance indicators such as financial outcomes, production lead times, or on-time delivery metrics. Finally, future studies may investigate moderating factors such as organisational culture, leadership orientation, and environmental uncertainty to deepen understanding of the contextual conditions that enhance or constrain the efficacy of digital transformation strategies.

## Supporting information

S1 Archive Supporting information. Data and results of instrument development.
(ZIP)
