# Supplementary material for: Stitching competition with digital threads: Unveiling the drivers of competitive success in the apparel sector
Source: PLoS One. 2025 Jun 10;20(6):e0325945. doi: 10.1371/journal.pone.0325945 (PMC12151375; doi:10.1371/journal.pone.0325945)
Supplement: Peer review history

# Author comment: Author response to Decision Letter 0

**DOI:** 10.1371/journal.pone.0325945.r001

27 Feb 2025

# Peer review: Decision Letter 0

**DOI:** 10.1371/journal.pone.0325945.r002

**Authors:** Manuel Herrador

Dear Dr. Susitha,

Thank you for submitting your manuscript to PLOS ONE. After careful consideration, we feel that it has merit but does not fully meet PLOS ONE’s publication criteria as it currently stands. Therefore, we invite you to submit a revised version of the manuscript that addresses the points raised during the review process.

Dear authors,

Based on the feedback from the reviewers, I recommend a major revision. Please address the points raised by the reviewers thoroughly. This revision will enhance the overall quality and impact of your work.

Thank you for your cooperation.

Best regards

Please submit your revised manuscript by Jun 05 2025 11:59PM. If you will need more time than this to complete your revisions, please reply to this message or contact the journal office at plosone@plos.org . When you're ready to submit your revision, log on to https://www.editorialmanager.com/pone/ and select the 'Submissions Needing Revision' folder to locate your manuscript file.

- A rebuttal letter that responds to each point raised by the academic editor and reviewer(s). You should upload this letter as a separate file labeled 'Response to Reviewers'.
- A marked-up copy of your manuscript that highlights changes made to the original version. You should upload this as a separate file labeled 'Revised Manuscript with Track Changes'.
- An unmarked version of your revised paper without tracked changes. You should upload this as a separate file labeled 'Manuscript'.

If you would like to make changes to your financial disclosure, please include your updated statement in your cover letter. Guidelines for resubmitting your figure files are available below the reviewer comments at the end of this letter.

If applicable, we recommend that you deposit your laboratory protocols in protocols.io to enhance the reproducibility of your results. Protocols.io assigns your protocol its own identifier (DOI) so that it can be cited independently in the future. For instructions see: https://journals.plos.org/plosone/s/submission-guidelines#loc-laboratory-protocols . Additionally, PLOS ONE offers an option for publishing peer-reviewed Lab Protocol articles, which describe protocols hosted on protocols.io. Read more information on sharing protocols at https://plos.org/protocols?utm_medium=editorial-email&utm_source=authorletters&utm_campaign=protocols .

We look forward to receiving your revised manuscript.

Kind regards,

Manuel Herrador, Ph.D.

Academic Editor

PLOS ONE

Journal Requirements:

1. When submitting your revision, we need you to address these additional requirements.

Please ensure that your manuscript meets PLOS ONE's style requirements, including those for file naming. The PLOS ONE style templates can be found at

https://journals.plos.org/plosone/s/file?id=wjVg/PLOSOne_formatting_sample_main_body.pdf and

https://journals.plos.org/plosone/s/file?id=ba62/PLOSOne_formatting_sample_title_authors_affiliations.pdf

2. We note that your Data Availability Statement is currently as follows: [All relevant data are within the manuscript and its Supporting Information files.]

Please confirm at this time whether or not your submission contains all raw data required to replicate the results of your study. Authors must share the “minimal data set” for their submission. PLOS defines the minimal data set to consist of the data required to replicate all study findings reported in the article, as well as related metadata and methods (https://journals.plos.org/plosone/s/data-availability#loc-minimal-data-set-definition).

For example, authors should submit the following data:

- The values behind the means, standard deviations and other measures reported;

- The values used to build graphs;

- The points extracted from images for analysis.

Authors do not need to submit their entire data set if only a portion of the data was used in the reported study.

If your submission does not contain these data, please either upload them as Supporting Information files or deposit them to a stable, public repository and provide us with the relevant URLs, DOIs, or accession numbers. For a list of recommended repositories, please see https://journals.plos.org/plosone/s/recommended-repositories.

If there are ethical or legal restrictions on sharing a de-identified data set, please explain them in detail (e.g., data contain potentially sensitive information, data are owned by a third-party organization, etc.) and who has imposed them (e.g., an ethics committee). Please also provide contact information for a data access committee, ethics committee, or other institutional body to which data requests may be sent. If data are owned by a third party, please indicate how others may request data access.

3. Please ensure that you include a title page within your main document. You should list all authors and all affiliations as per our author instructions and clearly indicate the corresponding author.

Additional Editor Comments:

Dear authors,

Based on the feedback from the reviewers, I recommend a major revision. Please address the points raised by the reviewers thoroughly. This revision will enhance the overall quality and impact of your work.

Thank you for your cooperation.

Best regards

[Note: HTML markup is below. Please do not edit.]

Reviewers' comments:

Reviewer's Responses to Questions

**Comments to the Author**

1. Is the manuscript technically sound, and do the data support the conclusions?

Reviewer #1: Yes

Reviewer #2: Yes

**********

2. Has the statistical analysis been performed appropriately and rigorously?

Reviewer #1: Yes

Reviewer #2: Yes

**********

3. Have the authors made all data underlying the findings in their manuscript fully available?

The PLOS Data policy

Reviewer #1: Yes

Reviewer #2: No

**********

4. Is the manuscript presented in an intelligible fashion and written in standard English?

Reviewer #1: Yes

Reviewer #2: Yes

**********

Reviewer #1: Overall paper looks good and however some of the literature has not been studied with respect to the digitization and metaverse in the fashion industry

Here is some suggestion that need to be addressed in the manuscript.

How can the validated measurement instrument be practically applied by apparel manufacturers to enhance their competitive performance?

How can apparel manufacturers practically apply the validated measurement instrument to enhance their competitive performance?

How do emerging technologies such as blockchain, IoT, and AI impact digital capabilities and competitive performance in the apparel industry?

Future research could explore the impact of emerging technologies on digital capabilities and competitive performance in the apparel industry.

Reviewer #2: General comments: This is an ambitious and timely study that offers a novel contribution to the measurement of digital capabilities and competitive performance in the apparel sector. The development of a validated measurement instrument is of high value to both academics and practitioners. The integration of qualitative and quantitative methods is well executed, and the authors present their findings in a structured and accessible manner.

Strengths: The article has sound theoretical grounding and clear research objectives, has a well-structured mixed-methods design, and makes use of appropriate and rigorous EFA and CFA. Furthermore, there is a strong alignment between qualitative findings and quantitative validation, and the article holds valuable practical implications for the apparel industry, especially in emerging markets.

That said, I have decided to accept the article with major revisions. The following are areas for improvement:

Broaden discussion of limitations: The limitations section should more directly address the geographical focus (Sri Lanka) and relatively small sample size, which may affect generalizability. Additionally, discuss potential cultural biases in the 8-point Likert scale based on Asian response patterns.

Clarify use of Orthogonal Rotation: Given that the constructs of CP and DC are conceptually related, it is important to justify the use of orthogonal (Varimax) over oblique rotation. If oblique rotation was tested and found inappropriate, please state so.

Improve clarity and reduce redundancy in language: Overall, the article would benefit from a thorough language edit to improve flow, grammar, and clarity. While the article may be intelligible, some parts read less polished or fluent. The manuscript contains redundancy, particularly in the abstract and results sections. A thorough copyedit is recommended to streamline the prose and ensure clarity. For example, remove repeated phrases such as “The study also developed a validated measurement instrument.”

Data Availability and transparency: While the data is said to be available in the manuscript and supporting files, the instrument or raw data (e.g., anonymised survey responses) would ideally be made available via an open-access repository to enhance replicability. Making the instrument and dataset available in a repository is now standard practice in open-access publishing. This improves reproducibility and reusability

Explain the theoretical and practical relevance of dimensions: The six validated dimensions are central to the paper, but their theoretical or practical significance is not sufficiently explored in the conclusion. How do these dimensions compare to existing frameworks in supply chain management or operations?

Consider broader application: Though the study is based in Sri Lanka, there may be potential for adapting the instrument to other apparel-producing countries. A brief discussion of this would improve the practical utility of the tool. Just a paragraph could add depth by commenting on adaptability or contextual factors in similar markets.

Theoretical framing: The paper would benefit from more explicit connection to existing frameworks (e.g., Resource-Based View, Dynamic Capabilities Theory), especially in discussing implications of the identified constructs.

**********

what does this mean? ). If published, this will include your full peer review and any attached files.

If you choose “no”, your identity will remain anonymous but your review may still be made public.

**Do you want your identity to be public for this peer review?** For information about this choice, including consent withdrawal, please see our Privacy Policy

Reviewer #1: **Yes: ** Aravin Prince Periyasamy

Reviewer #2: **Yes: ** Adil Boughlala

**********

[NOTE: If reviewer comments were submitted as an attachment file, they will be attached to this email and accessible via the submission site. Please log into your account, locate the manuscript record, and check for the action link "View Attachments". If this link does not appear, there are no attachment files.]

While revising your submission, please upload your figure files to the Preflight Analysis and Conversion Engine (PACE) digital diagnostic tool, https://pacev2.apexcovantage.com/ . PACE helps ensure that figures meet PLOS requirements. To use PACE, you must first register as a user. Registration is free. Then, login and navigate to the UPLOAD tab, where you will find detailed instructions on how to use the tool. If you encounter any issues or have any questions when using PACE, please email PLOS at figures@plos.org . Please note that Supporting Information files do not need this step.

# Author comment: Author response to Decision Letter 1

**DOI:** 10.1371/journal.pone.0325945.r003

19 May 2025

Authors’ Responses to Reviewer’s Comments – Manuscript Number: PONE-D-25-07767

Stitching competition with digital Threads: Unveiling the drivers of competitive success in the apparel sector

The authors express their sincere gratitude to the editors and reviewers for their meticulous examination and insightful feedback on our manuscript. Your expert comments and constructive suggestions have been invaluable in enhancing the quality of our work. We have diligently addressed each point raised, implementing necessary revisions to ensure our manuscript aligns with your esteemed standards. Enclosed, please find the authors' responses to the reviewer comments: the responses are presented in regular font, while the reviewer comments are distinctly italicised for ease of reference.

Response to Editor

Comment 1: Please ensure that your manuscript meets PLOS ONE's style requirements, including those for file naming.

Response: We appreciate the reviewer’s guidance. We have carefully reviewed the PLOS ONE submission guidelines and have revised the manuscript and all associated files to fully comply with the journal’s style requirements, including the appropriate file naming conventions.

Comment 2: We note that your Data Availability Statement is currently as follows: [All relevant data are within the manuscript and its Supporting Information files.]

Please confirm at this time whether or not your submission contains all raw data required to replicate the results of your study. Authors must share the “minimal data set” for their submission. PLOS defines the minimal data set to consist of the data required to replicate all study findings reported in the article, as well as related metadata and methods (https://journals.plos.org/plosone/s/data-availability#loc-minimal-data-set-definition).

For example, authors should submit the following data:

- The values behind the means, standard deviations and other measures reported;

- The values used to build graphs;

- The points extracted from images for analysis.

Authors do not need to submit their entire data set if only a portion of the data was used in the reported study.

If your submission does not contain these data, please either upload them as Supporting Information files or deposit them to a stable, public repository and provide us with the relevant URLs, DOIs, or accession numbers. For a list of recommended repositories, please see https://journals.plos.org/plosone/s/recommended-repositories.

Response: Thank you for your detailed guidance regarding the Data Availability Statement and minimal data set requirements. We confirm that we will upload all relevant data required to replicate the results of our study including the values behind reported statistics, figures, and analyses as Supporting Information files along with the final manuscript submission.

We will also ensure that the data files are formatted and labelled in accordance with PLOS ONE’s guidelines to facilitate transparency and reproducibility.

Comment 3: Please ensure that you include a title page within your main document. You should list all authors and all affiliations as per our author instructions and clearly indicate the corresponding author.

Response: We have included a title page in the main manuscript document, listing all authors with their respective affiliations in accordance with PLOS ONE’s author instructions. The corresponding author is clearly indicated as required.

Response to Reviewer 1

Comment 1: Overall paper looks good and however some of the literature has not been studied with respect to the digitization and metaverse in the fashion industry

Here is some suggestion that need to be addressed in the manuscript.

How can apparel manufacturers practically apply the validated measurement instrument to enhance their competitive performance?

How do emerging technologies such as blockchain, IoT, and AI impact digital capabilities and competitive performance in the apparel industry?

Future research could explore the impact of emerging technologies on digital capabilities and competitive performance in the apparel industry.

Response: We sincerely thank the reviewer for the constructive feedback and insightful suggestions, which have helped to further enhance the quality and relevance of our manuscript.

We have carefully addressed all the points raised:

1. We have expanded the literature review to include recent scholarly discussions on digitization and the emerging role of the metaverse within the fashion and apparel industry. This addition strengthens the theoretical foundation and contextual relevance of our study.

2. We have added a detailed explanation in the discussion and practical implications sections on how apparel manufacturers can use the validated measurement instrument to assess and enhance their competitive performance. Specific examples and strategic recommendations have been included to guide practical application.

3. We have integrated a discussion on how emerging technologies such as blockchain, IoT, and AI influence digital capabilities and, in turn, impact competitive performance. This discussion underscores the evolving digital landscape and its implications for the apparel supply chain.

4. As suggested, we have included a dedicated subsection outlining future research opportunities, particularly focusing on the impact of emerging technologies on digital transformation and competitive performance in the apparel industry.

Response to Reviewer 2

Comment 1: General comments: This is an ambitious and timely study that offers a novel contribution to the measurement of digital capabilities and competitive performance in the apparel sector. The development of a validated measurement instrument is of high value to both academics and practitioners. The integration of qualitative and quantitative methods is well executed, and the authors present their findings in a structured and accessible manner.

Response: We sincerely thank the reviewer for the encouraging and thoughtful feedback. We are pleased to know that the study’s contribution to measuring digital capabilities and competitive performance in the apparel sector has been recognized as both timely and novel. We greatly appreciate the recognition of the methodological rigor, particularly the integration of qualitative and quantitative approaches and the development of a validated measurement instrument.

Comment 2: Strengths: The article has sound theoretical grounding and clear research objectives, has a well-structured mixed-methods design, and makes use of appropriate and rigorous EFA and CFA. Furthermore, there is a strong alignment between qualitative findings and quantitative validation, and the article holds valuable practical implications for the apparel industry, especially in emerging markets.

Response: We sincerely thank the reviewer for the positive and encouraging feedback.

Comment 3: Broaden discussion of limitations: The limitations section should more directly address the geographical focus (Sri Lanka) and relatively small sample size, which may affect generalizability. Additionally, discuss potential cultural biases in the 8-point Likert scale based on Asian response patterns.

Response: We sincerely thank the reviewer for this valuable suggestion. In response, we have expanded the Limitations and Future Research Directions section to more directly address the geographical scope and methodological constraints of the study. Specifically, we now acknowledge that the focus on apparel manufacturers in Sri Lanka may limit the generalizability of the findings to other geographic regions or industrial sectors due to differences in institutional, cultural, and technological environments.

We have also highlighted the relatively small sample size as a limitation, noting that it restricts the statistical power and external validity of the results. This has been positioned as a justification for future studies to adopt larger and more diverse samples across multiple countries.

Additionally, we have incorporated a discussion on potential response bias related to the use of an 8-point Likert scale. In particular, we note that cultural response tendencies in Asian contexts such as moderate or socially desirable responding may influence how participants perceive and report constructs such as digital capabilities and performance.

Comment 4: Clarify use of Orthogonal Rotation: Given that the constructs of CP and DC are conceptually related, it is important to justify the use of orthogonal (Varimax) over oblique rotation. If oblique rotation was tested and found inappropriate, please state so.

Response: We thank the reviewer for this insightful observation. In response, we have clarified the rationale behind the use of orthogonal (Varimax) rotation in the EFA. Although the constructs of CP and DC are conceptually related, we initially tested both oblique (Promax) and orthogonal (Varimax) rotation methods. The results from both methods were comparable in terms of factor structure; however, the correlation matrix among the extracted factors in the oblique rotation was low (all correlations < 0.3), indicating minimal inter-factor correlation. Based on this, the orthogonal rotation was deemed appropriate for clearer factor interpretability and simplicity, without significant loss of conceptual rigor. We have included this justification in the revised methodology section.

Comment 5: Improve clarity and reduce redundancy in language: Overall, the article would benefit from a thorough language edit to improve flow, grammar, and clarity. While the article may be intelligible, some parts read less polished or fluent. The manuscript contains redundancy, particularly in the abstract and results sections. A thorough copyedit is recommended to streamline the prose and ensure clarity. For example, remove repeated phrases such as “The study also developed a validated measurement instrument.”

Response: We sincerely thank the reviewer for highlighting the need to enhance the clarity and fluency of the manuscript. In response, we have conducted a comprehensive language review and copyediting process to improve the overall readability, grammar, and academic tone. Particular attention was paid to removing redundant phrases.

We have revised these sections to streamline the prose, eliminate unnecessary repetition, and enhance the logical flow of arguments and findings. We trust that these improvements have strengthened the clarity and presentation of the manuscript, in line with the journal’s expectations.

Comment 6: Data Availability and transparency: While the data is said to be available in the manuscript and supporting files, the instrument or raw data (e.g., anonymised survey responses) would ideally be made available via an open-access repository to enhance replicability. Making the instrument and dataset available in a repository is now standard practice in open-access publishing. This improves reproducibility and reusability.

Response: We appreciate the reviewer’s valuable recommendation on enhancing transparency and replicability. In response, we confirm that we will make the full measurement instrument and anonymised raw survey data available through an open-access repository at the time of final submission. A link to the repository will be included in the revised Data Availability Statement. We fully support open-access publishing practices and agree that sharing these materials will strengthen the reproducibility and reusability of the study's findings.

Comment 7: Explain the theoretical and practical relevance of dimensions: The six validated dimensions are central to the paper, but their theoretical or practical significance is not sufficiently explored in the conclusion. How do these dimensions compare to existing frameworks in supply chain management or operations?

Response: Thank you for this insightful comment. In response, we have revised the Conclusion section to more clearly articulate the theoretical and practical significance of the six validated dimensions.

From a theoretical standpoint, we now discuss how these dimensions derived through rigorous empirical validation extend existing frameworks in supply chain management and operations by integrating both traditional and emerging performance drivers. Specifically, we highlight how the dimensions such as agility, customer satisfaction, and digital integration align with and expand upon well-established models like the RBV and the DCV, while also responding to recent calls in the literature for frameworks that incorporate digital transformation and real-time responsiveness in global supply chains.

From a practical perspective, we have elaborated on how these six dimensions offer apparel manufacturers a structured and actionable diagnostic tool to evaluate and enhance their competitive performance. Each dimension is discussed in terms of its relevance to decision-making, investment prioritization, and capability development in the context of fast-evolving, demand-driven markets particularly within emerging economies.

We believe these enhancements provide a clearer link between our findings and both the theoretical literature and real-world supply chain practices.

Comment 8: Consider broader application: Though the study is based in Sri Lanka, there may be potential for adapting the instrument to other apparel-producing countries. A brief discussion of this would improve the practical utility of the tool. Just a paragraph could add depth by commenting on adaptability or contextual factors in similar markets.

Response: We thank the reviewer for this constructive suggestion. In response, we have added a paragraph in the Discussion section addressing the potential for broader application of the validated measurement instrument beyond the Sri Lankan context. The revised text highlights how apparel manufacturers in other developing or export-driven economies may adapt the tool, while also acknowledging relevant contextual factors such as digital maturity, institutional infrastructure, and supply chain configuration. We believe this addition enhances the practical relevance and transferability of the instrument, aligning with the reviewer’s recommendation.

Comment 9: Theoretical framing: The paper would benefit from more explicit connection to existing frameworks (e.g., Resource-Based View, Dynamic Capabilities Theory), especially in discussing implications of the identified constructs.

Response: We appreciate the reviewer’s valuable suggestion regarding the need for a more explicit theoretical connection. In response, we have strengthened the theoretical framing throughout the manuscript particularly in the discussion and theoretical implications sections by explicitly linking the identified constructs to the RBV, DCV, and ERBV.

Attachment Submitted filename: Authors responses.docx

# Editor report: Decision Letter 1

**DOI:** 10.1371/journal.pone.0325945.r004

**Authors:** Manuel Herrador

<p>Stitching Competition with Digital Threads: Unveiling the Drivers of Competitive Success in the Apparel Sector

PONE-D-25-07767R1

Dear Dr. Susitha,

We’re pleased to inform you that your manuscript has been judged scientifically suitable for publication and will be formally accepted for publication once it meets all outstanding technical requirements.

Within one week, you’ll receive an e-mail detailing the required amendments. When these have been addressed, you’ll receive a formal acceptance letter and your manuscript will be scheduled for publication.

An invoice will be generated when your article is formally accepted. Please note, if your institution has a publishing partnership with PLOS and your article meets the relevant criteria, all or part of your publication costs will be covered. Please make sure your user information is up-to-date by logging into Editorial Manager at Editorial Manager®  and clicking the ‘Update My Information' link at the top of the page. If you have any questions relating to publication charges, please contact our Author Billing department directly at authorbilling@plos.org.

If your institution or institutions have a press office, please notify them about your upcoming paper to help maximize its impact. If they’ll be preparing press materials, please inform our press team as soon as possible -- no later than 48 hours after receiving the formal acceptance. Your manuscript will remain under strict press embargo until 2 pm Eastern Time on the date of publication. For more information, please contact onepress@plos.org.

Kind regards,

Manuel Herrador, Ph.D.

Academic Editor

PLOS ONE

Additional Editor Comments (optional):

Dear Authors,

I hope this message finds you well. I’m pleased to inform you that the reviewers who had initially requested major revisions are satisfied with the revisions.

Congratulations! Thanks for your contribution to PLOS One.

Best regards

Reviewers' comments:

# Editor report: Acceptance letter

**DOI:** 10.1371/journal.pone.0325945.r005

**Authors:** Manuel Herrador

PONE-D-25-07767R1

PLOS ONE

Dear Dr. Susitha,

I'm pleased to inform you that your manuscript has been deemed suitable for publication in PLOS ONE. Congratulations! Your manuscript is now being handed over to our production team.

At this stage, our production department will prepare your paper for publication. This includes ensuring the following:

* All references, tables, and figures are properly cited

* All relevant supporting information is included in the manuscript submission,

* There are no issues that prevent the paper from being properly typeset

You will receive further instructions from the production team, including instructions on how to review your proof when it is ready. Please keep in mind that we are working through a large volume of accepted articles, so please give us a few days to review your paper and let you know the next and final steps.

Lastly, if your institution or institutions have a press office, please let them know about your upcoming paper now to help maximize its impact. If they'll be preparing press materials, please inform our press team within the next 48 hours. Your manuscript will remain under strict press embargo until 2 pm Eastern Time on the date of publication. For more information, please contact onepress@plos.org.

If we can help with anything else, please email us at customercare@plos.org.

Thank you for submitting your work to PLOS ONE and supporting open access.

Kind regards,

PLOS ONE Editorial Office Staff

on behalf of

Dr. Manuel Herrador

Academic Editor

PLOS ONE
